# Pre-symptomatic radiological changes in frontotemporal dementia: propagation characteristics, predictive value and implications for clinical trials

**DOI:** 10.1007/s11682-022-00711-z

**Published:** 2022-08-03

**Authors:** Mary Clare McKenna, Jasmin Lope, Ee Ling Tan, Peter Bede

**Affiliations:** 1 Computational Neuroimaging Group, Biomedical Sciences Institute, Trinity College Dublin, Room 5.43, Pearse Street, Dublin 2, Ireland; 2 Department of Neurology, St James’s Hospital, Dublin, Ireland

**Keywords:** Frontotemporal dementia, MRI, Neuroimaging, Pre-symptomatic, Prodromal, Biomarker

## Abstract

Computational imaging and quantitative biomarkers offer invaluable insights in the pre-symptomatic phase of neurodegenerative conditions several years before clinical manifestation. In recent years, there has been a focused effort to characterize pre-symptomatic cerebral changes in familial frontotemporal dementias using computational imaging. Accordingly, a systematic literature review was conducted of original articles investigating pre-symptomatic imaging changes in frontotemporal dementia focusing on study design, imaging modalities, data interpretation, control cohorts and key findings. The review is limited to the most common genotypes: chromosome 9 open reading frame 72 (*C9orf72*), progranulin (*GRN*), or microtubule-associated protein tau (*MAPT*) genotypes. Sixty-eight studies were identified with a median sample size of 15 (3–141) per genotype. Only a minority of studies were longitudinal (28%; 19/68) with a median follow-up of 2 (1–8) years. MRI (97%; 66/68) was the most common imaging modality, and primarily grey matter analyses were conducted (75%; 19/68). Some studies used multimodal analyses 44% (30/68). Genotype-associated imaging signatures are presented, innovative study designs are highlighted, common methodological shortcomings are discussed and lessons for future studies are outlined. Emerging academic observations have potential clinical implications for expediting the diagnosis, tracking disease progression and optimising the timing of pharmaceutical trials.

**Supplementary Information:**

The online version contains supplementary material available at 10.1007/s11682-022-00711-z.

## Introduction

Frontotemporal dementia (FTD) incorporates a wide range of neurodegenerative disorders that present with diverse clinical phenotypes, radiological signatures, and underlying molecular pathology. A genetic cause is determined in approximately 30% of cases (Greaves & Rohrer, 2019). The most common genotypes include autosomal dominant mutations in chromosome 9 open reading frame 72 (*C9orf72*), progranulin (*GRN*), or microtubule-associated protein tau (*MAPT*) genes. In recent years, there have been concerted efforts to characterise the sequential cascade of clinical, imaging and biofluid alterations in the pre-symptomatic phase of familial FTD (Panman et al., 2021). These initiatives help to capture accruing disease-burden before it is clinically evident and imaging data provide additional insights on anatomical patterns of disease propagation. The practical aspiration of presymptomatic studies is to ascertain potential prognostic indicators, predict the clinical phenotype, forecast phenoconversion and suggest a window for viable therapeutic intervention. Given the increasing recognition of the clinical relevance of presymptomatic changes in familial FTD, the radiology literature of pre-symptomatic FTD is systematically reviewed.

## Methods

**Fig. 1:**
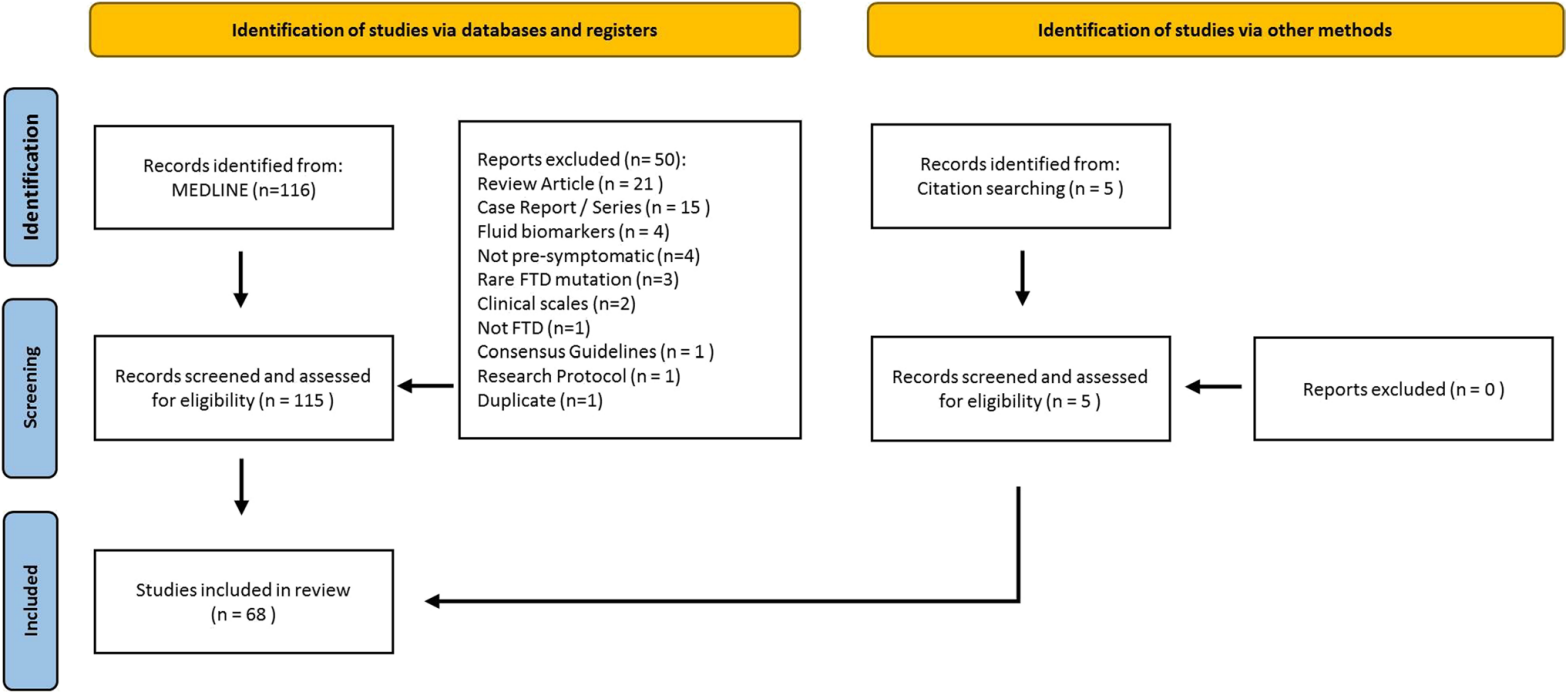
The flow diagram of the systematic review process

A systematic literature review was conducted using the MEDLINE database in accordance with the Preferred Reporting Items for Systematic Reviews and Meta-Analyses (PRISMA) recommendations. The core search terms ‘frontotemporal dementia’, ‘ FTD’, ‘frontotemporal lobar degeneration’ or ‘FTLD’ were individually combined with the keywords ‘pre-symptomatic’, ‘presymptomatic’, ‘asymptomatic’, ‘pre-clinical’, ‘prodromal’ or ‘pre-manifest’. This was followed by searching these pairings in combination with ‘magnetic resonance imaging’, ‘MRI’, ‘positron emission tomography’, ‘PET’, ‘MR spectroscopy’, ‘MRS’, ‘brain imaging’ or ‘neuroimaging’. The database search was limited to human studies written in English. It was last accessed in April 2022. Duplicate records were removed. A single reviewer individually screened and assessed the 116 records for eligibility. The inclusion criteria consisted of: original research papers that investigated pre-symptomatic radiological changes in the most common FTD genotypes: *C9orf72*, *GRN* and *MAPT*. Additional relevant records were identified from reference lists. Based on the above criteria a total of 68 eligible records were reviewed, grouped according to genotype and stratified according to imaging modality (Fig. 1).

## Results

Based on the above search criteria, 68 original research studies were identified that investigated pre-symptomatic radiological changes in *C9orf72*, *GRN* and *MAPT* mutation carriers (Fig. 1; Table 1). There were 26 studies that included more than one genotype; 15 studies investigated only *C9orf72* mutation carriers; 18 studies enrolled only *GRN* mutation carriers; and 9 studies evaluated only *MAPT* mutation carriers. The median (range) sample size for all genotypes was 15 (3–141); for *C9orf72* mutation carriers it was 28 (3–108); for *GRN* mutation carriers it was 32 (5–142); and for *MAPT* mutation carriers it was 13 (3–54). Only a minority of studies (28%) had a longitudinal design with a median (range) follow-up interval of 2 (1–8) years. Most of the studies relied on a single imaging modality (66%). The most common data acquisition technique was MRI (97%) that was interpreted in grey matter analyses (75%), white matter analyses (34%), functional analyses (29%) and spectroscopy (4%). There was a paucity of PET imaging studies (12%). Identified studies are first stratified according to the underlying genotype and then discussed from a methodological, academic and clinical viewpoint.

**Table 1:** Key study characteristics of presymptomatic neuroimaging initiatives in familial FTD/FTLD

	All genotypes	*MAPT*	*C9orf72*	*GRN*
Reviewed Studies	68	34	37	43
% of studies from database	100% (68/68)	50% (34/68)	54% (37/68)	63% (43/68)
Sample size per genotype—Average	23	18	36	47
Sample size per genotype—Median	15	13	28	32
Sample size per genotype—Range	(3–141)	(3–54)	(3–108)	(5–142)
Longitudinal	28% (19/68)	30% (10/34)	30% (11/37)	28% (12/43)
Follow-up – Average (years)	2.6	3.5	2	2.7
Follow-Up – Median (years)	2	3	1.5	2
Follow up – Range (years)	(1–8)	(1–8)	(1–6)	(1–6)
Multimodal % (n)	44% (30/68)	29% (10/34)	35% (13/37)	42% (18/43)
MRI % (n)	97% (66/68)	97% (33/34)	97% (36/37)	100% (43/43)
Grey Matter Analyses % (n)	75% (51/68)	68% (23/34)	81% (29/36)	81% (35/43)
White Matter Analyses % (n)	34% (23/68)	26% (9/34)	33% (12/36)	30% (13/43)
Functional MRI % (n)	29% (20/68)	29% (10/34)	25% (9/36)	37% (16/43)
MR Spectroscopy % (n)	4% (3/68)	9% (3/34)	0% (0/36)	0% (0/43)
PET % (n)	12% (8/68)	9% (3/34)	8% (3/37)	5% (2/43)

## *C9orf72*

The majority of radiological studies of pre-symptomatic *C9orf72* GGGGCC repeat expansion carriers describe widespread structural and functional changes. It remains debated whether such findings represent neurodevelopmental or neurodegenerative change given the early onset and relatively slow progression (Lulé et al., 2020a). It has been proposed that radiological changes may begin in the thalamus and posterior cortical regions, later involving the frontotemporal regions, and may be identified up to 25 years before symptom onset (Rohrer et al., 2015). Preliminary multimodal MRI classification models have shown that individual radiological changes may not be evident until a few years before symptom onset in pre-symptomatic FTD mutation carriers (Feis et al., 2019). We next discuss the evidence of pre-symptomatic radiological changes in *C9orf72* repeat expansion mutation carriers (Supplementary Table 1).

Widespread cortical and subcortical grey matter (GM) pathology is often detected, but may be too subtle for visual detection (Fumagalli et al. 2018). Cortical thinning is observed in the frontal (Popuri et al., 2018; Blanc et al., 2020; Panman et al., 2019), temporal (Popuri et al., 2018; Blanc et al., 2020; Walhout et al., 2015), parietal (Popuri et al., 2018; Blanc et al., 2020; Panman et al., 2019; Walhout et al., 2015), and occipital cortices (Walhout et al., 2015). Volume loss is relatively symmetrical (Bocchetta et al., 2021), involving the frontal (Rohrer et al., 2015; Panman et al., 2019; Bocchetta et al., 2021; Bertrand et al., 2018; Lee et al., 2016; Olney et al., 2020; Russell et al., 2020; Cash et al., 2018), temporal (Rohrer et al., 2015; Panman et al., 2019; Bocchetta et al., 2021; Bertrand et al., 2018; Olney et al., 2020; Russell et al., 2020; Cash et al., 2018; Papma et al., 2017), parietal (Panman et al., 2019; Bocchetta et al., 2021; Bertrand et al., 2018; Cash et al., 2018; Papma et al., 2017), insular (Rohrer et al., 2015; Panman et al., 2019; Bocchetta et al., 2021; Lee et al., 2016; Olney et al., 2020; Russell et al., 2020), cerebellar (Rohrer et al., 2015; Panman et al., 2019; Bocchetta et al., 2021; Cash et al., 2018; Papma et al., 2017; McKenna et al., 2021a) regions. Relatively selective cerebellar involvement has been suggested by some (McKenna et al., 2021a) with the preferential degeneration of lobules VIIa, VIIb, Crus I and II (Bocchetta et al., 2021; Cash et al., 2018). In an admixed group of pre-symptomatic FTD mutation carriers, there is also early change in ventricular volume compared to controls (Tavares et al., 2019). Subcortical (Popuri et al., 2018; Walhout et al., 2015; Bocchetta et al., 2021; Bertrand et al., 2018; Lee et al., 2016; Papma et al., 2017) degeneration has been recently further characterised by reports of preferential degenerative change in specific subcortical sub-regions. Focal thalamic changes (Rohrer et al., 2015; Popuri et al., 2018; Panman et al., 2019; Bocchetta et al., 2021; Bertrand et al., 2018; Lee et al., 2016; Olney et al., 2020; Cash et al., 2018; Papma et al., 2017; Cury et al., 2019; McKenna et al., 2022) have been described in the anterior (Cury et al., 2019; McKenna et al., 2022), laterodorsal (Bocchetta et al., 2021), lateral geniculate nuclei (Bocchetta et al., 2021) as well as in pulvinar regions (Bocchetta et al., 2021). Preferential caudate (Popuri et al., 2018; Walhout et al., 2015; Russell et al., 2020), putamen (Walhout et al., 2015; Bocchetta et al., 2021; Russell et al., 2020), amygdala (Bocchetta et al., 2021; Russell et al., 2020; Chipika et al., 2020) and hypothalamus (Bocchetta et al., 2021) pathology has also been described. In some studies degenerative changes were only detected in older cohorts aged > 40 years (Papma et al., 2017). This trend of progressive changes in older subgroups was shown in a study that described widespread changes in pre-symptomatic cohorts aged > 40 years compared with those aged < 40 years (Bertrand et al., 2018). The rate of cortical thinning has been calculated as either faster (Blanc et al., 2020) or no different (Waugh et al., 2021) compared to controls. Patterns of atrophy have been evaluated (Olney et al., 2020) to predict phenoconversion (Staffaroni et al., 2020). The level of educational attainment (Gazzina et al., 2019; Premi et al., 2017) and *TMEM106B* genotype (Premi et al., 2017) are considered to be modifying factors. Some pre-symptomatic structural changes are thought to be associated with early behavioral changes; apathy has been linked to frontal and cingulate pathology (Malpetti et al., 2021a); and impaired social cognition to insula, basal ganglia, amygdala, and frontotemporal involvement (Russell et al., 2020). In addition to standard morphometric and volumetric GM methods, a number of novel analysis pipelines have also been implemented. Early abnormal gyrification index has been described in the left anterior cingulate cortex, left precentral gyrus, right inferior parietal, and right superior occipital regions decades before expected symptom onset (Caverzasi et al., 2019). This anatomical pattern is similar to the focal regions of atrophy described in both pre-symptomatic and symptomatic cases, despite no corresponding cortical thickness abnormalities detected in this study (Caverzasi et al., 2019). Neurite orientation dispersion and density imaging (NODDI) also detected more widespread GM abnormalities in frontal, temporal parietal, occipital and insular regions compared to conventional volumetric measures (Wen et al., 2019). Reduced cortical surface area has been described in a similar but more restricted anatomical distribution to symptomatic cohorts, particularly in the ventrofrontal regions (Blanc et al. 2020). It is noteworthy that a minority of published studies do not detect any pre-symptomatic GM pathology (Waugh et al., 2021; Floeter et al., 2016; Popuri et al., 2021; Convery et al., 2020).

Widespread WM degeneration has been repeatedly described in pre-symptomatic *C9orf72* repeat expansion carriers typically involving the corpus callosum (Bertrand et al., 2018; Lulé et al., 2020b), thalamic radiation (Panman et al., 2019; Bertrand et al., 2018; Papma et al., 2017), uncinate fasciculus (Lee et al., 2016), superior longitudinal fasciculus (Panman et al., 2019), inferior longitudinal fasciculus (Lee et al., 2016), corticospinal tracts (Panman et al., 2019; Lee et al., 2016; Querin et al., 2019a), orbitofrontal regions (Lulé et al., 2020b) and other frontal WM tracts (Panman et al., 2019; Lee et al., 2016; Papma et al., 2017). These structural changes may be associated with incipient executive dysfunction, specifically reduced verbal fluency (Lulé et al., 2020b). It is proposed that WM pathology may precede or occur in tandem with GM degeneration (Bertrand et al., 2018; Papma et al., 2017; Lulé et al., 2020b; Querin et al., 2019a). Recent MRI classification models in pre-symptomatic FTD mutation carriers indicate that the earliest radiological changes occur in the WM because WM features offer the best discriminating value from controls (Feis et al., 2019). Longitudinal studies have shown strikingly inconsistent results depending on cohort and region of interest (ROI) characteristics. In pre-symptomatic *C9orf72* carriers aged > 40 years, significant baseline cervical spinal cord WM atrophy was described, with ensuing corticospinal tract (CST) FA reductions on interval imaging over an 18-month period (Querin et al., 2019a). In contrast, no significant progression of brain imaging changes were identified over a 12-month follow-up period (Lulé et al., 2020b). Similar to GM analyses, novel WM methods have also been increasingly implemented. Neurite orientation dispersion and density imaging (NODDI) readily detects corticospinal and frontotemporal WM tracts abnormalities with greater sensitivity than standard diffusivity metrics in pre-symptomatic *C9orf72* cohorts (Wen et al., 2019). A minority of studies do not detect any pre-symptomatic diffusivity abnormalities (Waugh et al., 2021). However, subtle internal capsule (IC) and the corpus callosum (CC) changes may be detected on longitudinal follow-up in some of these studies (Waugh et al., 2021). The pre-symptomatic phase of *C9orf72* is not thought to be associated with increased WM hyperintensity burden (Sudre et al., 2017).

Functional imaging changes are also evident several years before symptom onset (Lee et al., 2016; Popuri et al., 2021; Premi et al., 2019; Rittman et al., 2019), sometimes preceding the detection of structural imaging abnormalities (Waugh et al., 2021; Popuri et al., 2021). [^18^F] FDG-PET studies demonstrate significant frontotemporal hypometabolism in the insular cortex, central opercular cortex, basal ganglia and thalami (Popuri et al., 2021; Vocht et al., 2020), with the additional involvement of the inferior parietal lobes and adjacent regions (Popuri et al., 2021). A [^11^ C]UCB-J PET study has shown pre-symptomatic synaptic density reduction in the thalamus that was most marked in pulvinar and ventral-posterior regions with progressive cortical and subcortical loss of synaptic density (Malpetti et al., 2021b). Preliminary studies using arterial spin labelling (ASL) have described cerebral hypoperfusion in the insula, orbitofrontal, anterior cingulate, temporal and inferior parietal cortices up to 12.5 years before expected symptom onset (Mutsaerts et al., 2019). Functional connectivity alterations have also been described (Lee et al., 2016; Waugh et al., 2021) that may (Waugh et al., 2021) or may not (Lee et al., 2016) occur with associated structural changes. A longitudinal study described increased sensorimotor network connectivity adjacent to regions which later become affected in symptomatic cohorts (Waugh et al., 2021). In contrast, reduced functional connectivity has been described in thalamic, frontotemporal and motor networks in a less extensive but similar anatomical distribution to symptomatic cohorts (Shoukry et al., 2020). It is hypothesised that the maintenance of functional network topography facilitates cognitive resilience in face of relentless structural changes (Rittman et al., 2019; Tsvetanov et al., 2021). The integrity of these functional networks then rapidly declines as patients become symptomatic (Rittman et al., 2019).

### *GRN*

In pre-symptomatic *GRN* mutation carriers, there is ample radiological evidence of structural and functional alterations, typically involving frontal, parietal and subcortical regions in a similar but more restricted pattern to symptomatic cases (Pievani et al., 2014). These findings may be evident several years before symptom onset, but may be very subtle or elude detection for a variety of reasons that are later discussed. They are best detected in mutation carriers who are approaching the expected age of phenoconversion (Jiskoot et al., 2019). Herein we summarise the observed pre-symptomatic radiological findings (Supplementary Table 2).

In pre-symptomatic *GRN* mutation carriers, several studies report no difference in cortical or subcortical volumes compared to controls (Popuri et al., 2018; Panman et al., 2019; Bocchetta et al., 2021; Borroni et al., 2008, 2012; Borrego-Écija et al., 2021; Feis et al., 2019; Premi et al., 2016; Lee et al., 2019). The ability to detect GM pathology may depend on the interval to projected phenoconversion (Fumagalli et al., 2018; Cash et al., 2018) and subtle changes may require longitudinal follow-up for detection (Caroppo et al., 2015). GM degeneration is typically not appreciated on visual rating scales (Fumagalli et al., 2018). GM volume loss is thought to first occur in insular regions (Panman et al., 2021; Rohrer et al., 2015; Cash et al., 2018; Gazzina et al., 2018; Olm et al., 2018) up to 15-years before symptom onset (Rohrer et al., 2015); followed by frontal (Cash et al., 2018; Pievani et al., 2014; Olm et al., 2018; Chen et al., 2020), parietal (Rohrer et al., 2015; Olney et al., 2020; Cash et al., 2018; Gazzina et al., 2018), temporal (Panman et al., 2021; Rohrer et al., 2015; Olney et al., 2020; Cash et al., 2018; Caroppo et al., 2015; Olm et al., 2018), occipital (Chen et al., 2020) and subcortical atrophy (Rohrer et al., 2015; Cash et al., 2018). Frontal lobe changes typically involve orbitofrontal (Pievani et al., 2014; Olm et al., 2018) and posterior (Cash et al., 2018) regions; these early alterations may be associated with progressive apathy (Malpetti et al., 2021a). The temporal lobe alterations may be predominantly anterior (Cash et al., 2018; Olm et al., 2018), posterior (Olney et al., 2020), and lateral (Caroppo et al., 2015). Longitudinal studies have detected the greatest rate of atrophy in the pre-symptomatic phase in the frontal (Olm et al., 2018; Chen et al., 2020), parietal (Chen et al., 2020) and occipital (Olm et al., 2018) lobes. Characteristic asymmetry (Rohrer et al., 2015) and differences in ventricular volumes (Tavares et al., 2019) may be detected a few years before symptom onset. Pre-symptomatic subcortical changes are also readily detected in *GRN* mutation carriers. Anterior thalamic shape deformation was described at least 5-years before symptom onset (Cury et al., 2019). The thalamus and basal ganglia have both been implicated in an admixed group of pre-symptomatic and symptomatic *GRN* mutation carriers (Russell et al., 2020). The characterisation of atrophy patterns may be used to discriminate pre-symptomatic and symptomatic FTD mutation carriers (Staffaroni et al., 2020). The degree of GM volume loss may be influenced by level of educational attainment (Gazzina et al., 2019; Premi et al., 2017), which is further modulated by the TMEM106B genotype (Premi et al., 2017). Other modifiers include high leukocyte mRNA levels of inflammation-related TMEM40 and LY6G6F that are associated with greater parietal and superior frontal lobe atrophy respectively (Milanesi et al., 2013).

Pre-symptomatic *GRN* mutation carriers also exhibit extensive WM degeneration (Jiskoot et al., 2019) which may be evident several years before symptom onset (Pievani et al., 2014) and rapidly progresses prior to phenoconversion (Jiskoot et al., 2019). The loss of WM integrity detected by diffusivity metrics typically involves the corpus callosum (Jiskoot et al., 2019; Olm et al., 2018), superior longitudinal fasciculus (Panman et al., 2021; Pievani et al., 2014; Olm et al., 2018), corticospinal tracts (Pievani et al., 2014; Olm et al., 2018), the cingulum (Pievani et al., 2014), uncinate (Panman et al., 2021; Borroni et al., 2008) and inferior occipitofrontal fasciculi (Borroni et al., 2008). There is progressive WM degeneration that is maximal in the genu of the corpus callosum (Jiskoot et al., 2019; Olm et al., 2018) and the right-sided superior longitudinal fasciculus (Olm et al., 2018) in the 2-years prior to symptom onset (Jiskoot et al., 2019). Patterns of preferential WM vulnerability depend on the subsequent clinical phenotype, with early involvement of the uncinate fasciculus in non-fluent primary progressive aphasia (nfvPPA) and of the superior longitudinal fasciculus in behavioral variant FTD (bvFTD) (Panman et al., 2021). There thought to be an increased burden of WM hyperintensities (Sudre et al., 2019) that accumulate over time, particularly in the periventricular frontal, parietal and occipital regions (Sudre et al., 2017; Sudre et al., 2019). These WM hyperintensities have been linked to executive dysfunction, *TMEM106B* risk genotype, low GM volume, and elevated neurofilament light chains (Sudre et al., 2019). The sequential order of radiological changes is yet to be determined. Some studies suggest that WM degeneration precedes GM degeneration (Panman et al., 2021; Feis et al., 2019); while other studies suggest that it occurs simultaneously (Jiskoot et al., 2019). The best-performing multimodal MRI classification models use exclusively WM features to categorise individual pre-symptomatic mutation carriers (McKenna et al., 2022), highlighting the superior specificity of WM signatures (Feis et al., 2019). This is further supported by data-driven disease progression modelling initiatives that relied on cross-sectional data to estimate the cascade of biomarkers and suggest that WM diffusivity abnormalities preceded GM loss, and that the left hemisphere is involved before the right hemisphere (Panman et al. 2021). These diffusivity abnormalities however are typically only detected 2–4 years prior to symptom onset (Jiskoot et al., 2019). This may explain why some studies do not detect any WM diffusivity alterations (Panman et al., 2019; Feis et al., 2019), WM volume loss (Borroni et al., 2008) or WM hyperintensities (Sudre et al., 2017) in pre-symptomatic *GRN* mutation carriers. As a consequence, MRI-based classification scores often remain similar to controls until approaching phenoconversion (Feis et al., 2019).

Pre-symptomatic functional imaging changes have also been described (Premi et al., 2019). In [^18^F] FDG-PET studies, asymmetric cerebral hypometabolism is typically reported involving either the left (Caroppo et al., 2015) or right (Jacova et al., 2013) hemisphere – primarily localised to the frontal (Caroppo et al., 2015; Jacova et al., 2013), insular (Jacova et al., 2013) or temporal (Caroppo et al., 2015) lobes. Regional cerebral hypometabolism is thought to precede structural imaging changes and may be detected up to 20-years before expected symptom onset (Caroppo et al., 2015). Studies using arterial spin labelling (ASL), a non-invasive method of quantifying cerebral perfusion, have demonstrated reduced cerebral blood flow in frontal, temporal, parietal and subcortical regions in pre-symptomatic FTD mutation carriers up to 12.5 years before expected symptom onset (Mutsaerts et al., 2019; Dopper et al., 2016). In pre-symptomatic *GRN* mutation carriers, asymmetric frontoparietal hypoperfusion involving the bilateral anterior cingulate/paracingulate, right anterior insula/orbitofrontal, and right supramarginal/angular gyri has been reported (Mutsaerts et al., 2019; Dopper et al., 2016). Functional connectivity deficits have also been repeatedly described involving the frontal (Premi et al. 2016; Premi et al. 2014), parietal (Premi et al. 2016; Premi et al. 2014), and thalamic (Lee et al. 2019) regions which may also precede structural deficits (Premi et al., 2016; Lee et al., 2019). Both decreased and increased functional connectivity have been reported depending on the age profile, education and definition of seed regions. Cognitive reserve is also an important modifying factor (Premi et al., 2013; Costello et al., 2021) which should be considered in the interpretation of clinico-radiological correlations. Altered dynamic functional connectivity with increased activation of the insula and parietal regions has been recently reported (Premi et al., 2021). Initial hyperconnectivity involving the salience (Borroni et al., 2012; Lee et al., 2019), default mode (Lee et al., 2019), perirolandic (Lee et al., 2019) and language networks (Lee et al., 2019) has been described. The latter was asymmetric with progressively reducing connectivity with age (Lee et al., 2019). Other studies identified reduced salience network connectivity (Premi et al., 2013). It remains unclear whether increased connectivity represents a compensatory mechanism (Lee et al., 2019) reduced inhibition or stems from methodological factors (Proudfoot et al., 2018). Some studies suggest that the maintenance of functional network organisation contributes to cognitive resilience in face of evolving structural degeneration (Rittman et al., 2019; Tsvetanov et al., 2021; Bede et al., 2021a). The subsequent loss of functional network organisation is associated with emergent cognitive symptoms (Rittman et al., 2019; Tsvetanov et al., 2021). While some studies detect complex functional reorganisation, others do not detect functional connectivity alterations (Premi et al., 2019; Pievani et al., 2014; Feis et al., 2019; Premi et al., 2021).

### *MAPT*

Pre-symptomatic *MAPT* mutation carriers exhibit evidence of insidious radiological involvement, typically beginning in the medial temporal lobes, extending to the insula and accelerating 2-years before symptom onset (Rohrer et al., 2015; Panman et al., 2019; Jiskoot et al., 2019; Dopper et al., 2016; Chen et al., 2019a). Multimodal MRI based classification models suggest that individual radiological changes may not detectable until a few years before phenoconversion (Feis et al., 2019; McKenna et al., 2022). Evidence for pre-symptomatic radiological changes in *MAPT* mutation carriers is summarized in Supplementary Table 3.

Pre-symptomatic cortical (Rohrer et al., 2015; Panman et al., 2019) and subcortical (Rohrer et al., 2015; Cury et al., 2019) GM pathology may be detected up to 15-years before symptom onset. In the pre-symptomatic phase, cortical changes may be detected in the insula, anterior cingulate, orbitofrontal and medial temporal regions (Rohrer et al., 2015; Panman et al., 2019; Olney et al., 2020; Cash et al., 2018; Clarke et al., 2021). Medial temporal lobe atrophy may even be detected by visual inspection using visual rating scales (Fumagalli et al., 2018). In pre-symptomatic FTD mutation carriers, there is also a difference in ventricular volume (Tavares et al., 2019). In the minimal and mild symptomatic phase, GM degeneration extends to involve the dorsolateral temporal cortex (Bocchetta et al., 2021), cingulate cortex and lingual gyrus in the occipital lobe (Domínguez-Vivero et al., 2020). This peri-symptomatic involvement of the cingulate cortices has been linked to progressive apathy (Malpetti et al., 2021a). Subcortical involvement has been described in the anterior thalamus in an admixed group of pre-symptomatic FTD mutation carriers (Cury et al., 2019). Amygdalar (Rohrer et al., 2015; Bocchetta et al., 2021) and hippocampal pathology (Rohrer et al., 2015; Panman et al., 2019; Bocchetta et al., 2021; Miyoshi et al., 2010) have been detected in a subgroup of pre-symptomatic *MAPT* carriers, but this is not a universal finding (Kantarci et al., 2010). However, significant differences may only be detected if the volumes of specific subregions are estimated rather than considering the overall volume of the entire structure. For instance, the selective involvement of the accessory basal and superficial nuclei subregions of the amygdala may be detected before the total volume of the amygdala changes (Bocchetta et al., 2021). In pre-symptomatic FTD mutation carriers, the quantification of individual GM patterns may be used to predict disease progression (Staffaroni et al., 2020; McKenna et al., 2022). Level of educational attainment (Gazzina et al., 2019; Premi et al., 2017) and *TMEM106B* genotype (Premi et al., 2017) are considered individual modifying factors. In some studies, GM pathology is not detected for a variety of reasons that are later discussed (Bocchetta et al., 2021; Feis et al., 2019; Dopper et al., 2014).

There is also evidence for genotype-specific patterns of WM degeneration involving the frontotemporal tracts (Dopper et al., 2014). A longitudinal study of pre-symptomatic *MAPT* mutation carriers demonstrated entorhinal WM pathology that extended into the limbic and frontotemporal projections after phenoconversion (Chen et al., 2019b). Loss of WM integrity has also been described in the bilateral uncinate fasciculus, left anterior thalamic radiation, left inferior fronto-occipital fasciculus (Panman et al., 2019; Dopper et al., 2014) evolving 2-years before phenoconversion, but not earlier than this (Jiskoot et al., 2019). While the involvement of the uncinate fasciculus is not unique to this genotype, it was more markedly involved in pre-symptomatic *MAPT* mutation carriers compared to *GRN* mutation carriers (Jiskoot et al., 2019). In contrast, another study only found uncinate involvement in symptomatic cases (Chen et al., 2019b). The chronology of sequential GM and WM pathology is not well defined. Multimodal MRI classification studies indicate that the earliest pre-symptomatic changes are WM alterations in FTD mutation carriers (Feis et al., 2019); whereas other studies suggest simultaneous GM and WM pathology, with predominant loss of WM integrity (Jiskoot et al. 2019). Conversely, frank diffusivity abnormalities may not be readily identified in pre-symptomatic *MAPT* mutation carriers (Jiskoot et al., 2019; Feis et al., 2019; Domínguez-Vivero et al., 2020) and no marked WM hyperintensity burden has been detected (Sudre et al., 2017).

MR spectroscopy studies have suggested a relatively stereotyped sequence of events, beginning with increased mI/Cr ratio (indicators of glial activity), followed by decreased NAA/mI ratio (markers of loss of neuronal integrity) and subsequent atrophy (Christidi et al., 2022). MRS studies of presymptomatic *MAPT* mutation carriers are predominantly single voxel studies focusing on different regions of interests (ROIs) such as the posterior cingulate gyrus inferior precuneus (Chen et al., 2019a; Kantarci et al., 2010) or medial frontal lobe (Chen et al., 2019c). Similar to structural findings, these radiological changes accelerate in the 2-years preceding symptom onset (Chen et al., 2019a). Cross-sectional studies have reported divergent results of NAA/Cr ratios: some studies have demonstrated decreased NAA/Cr ratios in the medial frontal lobe (Chen et al., 2019c); and other studies have shown no difference in the posterior cingulate gyrus inferior precuneus (Kantarci et al., 2010). Given that decreased NAA/Cr ratio is a relatively consistent finding in symptomatic *MAPT* mutation carriers, these findings may signal impending phenoconversion (Kantarci et al., 2010).

Pre-symptomatic PET studies have used different radiotracers. An [^18^F] flortaucipir PET study showed slightly elevated binding in the insula, frontal, parietal and medial temporal lobe indicating tau pathology (Wolters et al., 2021), A multi-modal PET study showed dopaminergic dysfunction in the putamen using l-[β-^11^C]dopa PET, and variable levels of glial activation using [^11^C]DAA1106 PET in the frontal, occipital and posterior cingulate cortices (Miyoshi et al., 2010). An [^18^F] FDG-PET study demonstrated anterior cingulate hypometabolism (Clarke et al., 2021). Studies using arterial spin labelling have detected a trend of relatively symmetrical perfusion reduction in the frontal and subcortical areas in *MAPT* mutation carriers (Dopper et al., 2016) up to 12.5 years before expected symptom onset (Mutsaerts et al., 2019). fMRI studies have supported the notion of accruing radiological findings prior to phenoconversion (Premi et al., 2019; Dopper et al., 2016). Altered functional connectivity has been reported in the default mode network preceding structural atrophy (Whitwell et al., 2011). It has been repeatedly proposed that preserved functional network integrity enables cognitive resilience in the setting of pre-symptomatic functional and structural radiological abnormalities (Rittman et al., 2019; Tsvetanov et al., 2021). It is noteworthy however that pre-symptomatic functional connectivity alterations may not readily detected in *MAPT* mutation carriers (Feis et al., 2019; Dopper et al., 2014).

## Discussion

**Fig. 2:**
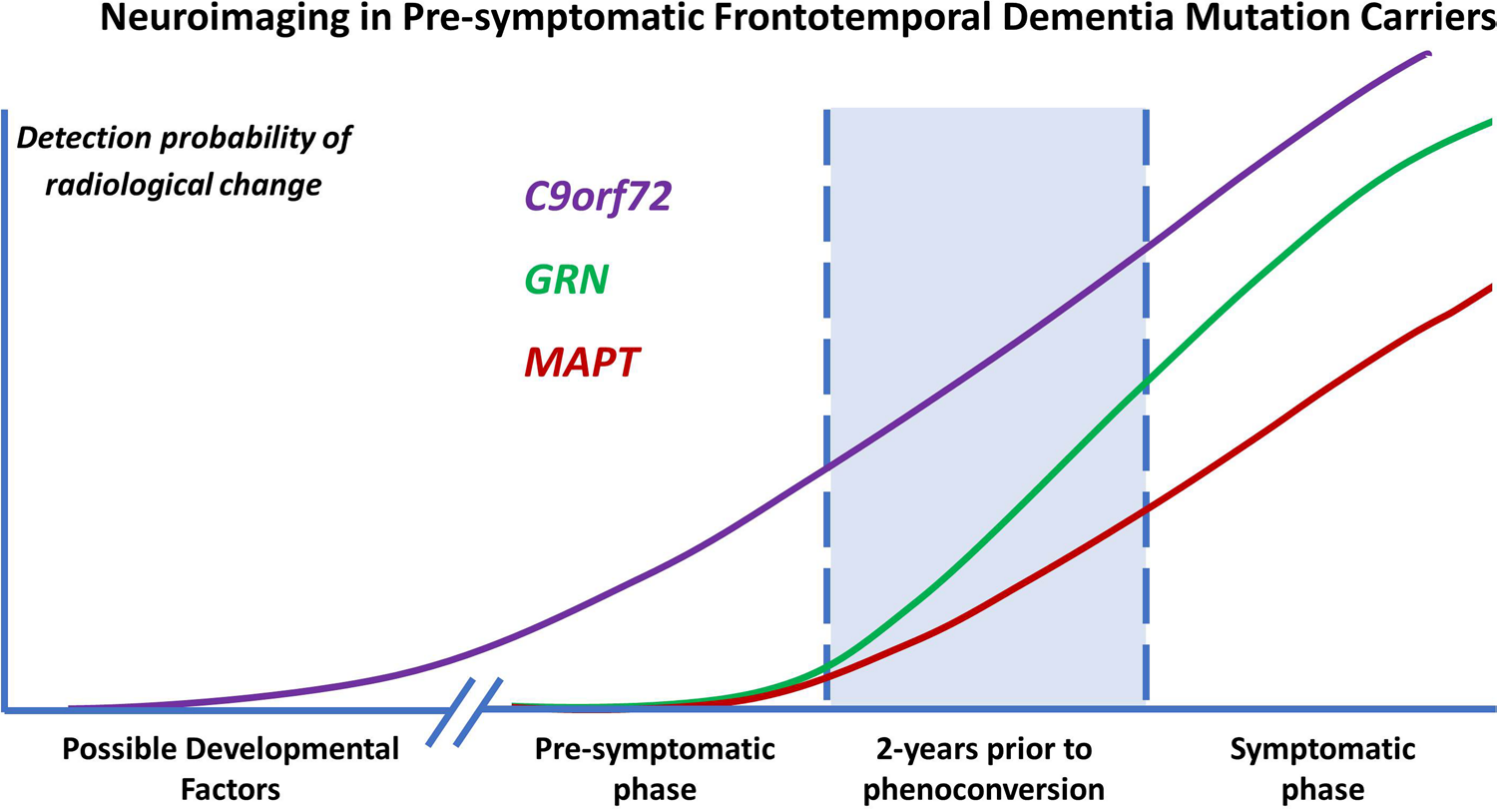
A schematic representation of the detection likelihood of presymptomatic radiological change in the most common FTD-associated genetic variants. In *C9orf72* mutation carriers, it is hypothesized that neurodevelopmental factors may be at play in conjunction with slowly progressive neurodegeneration. In *GRN* mutation carriers, the disease process is thought to accelerate 2-years before phenoconversion. In *MAPT* mutation carriers, disease burden accrues 2-years before phenoconversion, but at a relatively slower rate than in *GRN*

There is a consensus in the literature that pre-symptomatic structural and functional imaging changes may be detected in *C9orf72*, *GRN*, and *MAPT* mutation carriers several years before expected symptom onset, which become particularly marked in the period leading up to phenoconversion. A multitude of imaging methods has been successfully implemented in presymptomatic gene carriers and the various modalities not only offer complementary information but are relatively consistent with regards to anatomical patterns of preferential vulnerability. Despite considerable methodological differences, focus on diverse ROIs, and divergent cohort characteristics, consensus study findings can be identified. In pre-symptomatic *C9orf72* mutation carriers, there is widespread cortical and subcortical GM involvement beginning in the thalamus and posterior cortical regions, gradually involving the frontotemporal regions. This is coupled with extensive WM degeneration, frontotemporal hypometabolism and altered functional connectivity involving thalamic, frontotemporal and motor networks. In pre-symptomatic *GRN* mutation carriers, there is relatively asymmetric cortical and subcortical GM pathology often spreading from insular regions, gradually involving frontal, parietal, temporal and thalamic brain regions. There is also extensive WM degeneration in particular at the genu of the corpus callosum, increased WM hyperintensity burden, asymmetric frontotemporal hypometabolism and altered functional connectivity in thalamic, frontal and parietal circuits. In pre-symptomatic *MAPT* mutation carriers, there is more focal GM involvement centred on the medial temporal lobe, later involving the insula and frontal regions. WM degeneration in pre-symptomatic *MAPT* is particularly marked in the uncinate fasciculus. There is also ample evidence of frontal and subcortical hypometabolism and altered functional connectivity involving frontal networks. Multi-parametric imaging studies also offer insights regarding the likely chronology of radiological changes and biological cascades preceding phenoconversion (Fig. 2). While there is no definite consensus on a specific timeline, there are indications of early metabolic and functional changes followed by structural degeneration before symptom onset. Classification studies have consistently highlighted that WM features best discriminate pre-symptomatic mutation carriers from controls suggesting that WM alterations are relatively specific and early radiological features.

It is increasingly debated whether pre-symptomatic radiological changes, particularly in *C9orf72* mutation carriers, may represent early neurodegeneration or neurodevelopmental abnormalities (Lulé et al., 2020a; Bede et al., 2020). In favour of neurodegeneration, *C9orf72* mutation carriers exhibit a slowly evolving progressive radiological profile that is considered to represent the insidious pathological process several decades before symptom onset (Bocchetta et al., 2021; Bertrand et al., 2018). Moreover, there are well described patterns and stages of pTDP-43 pathology in *C9orf72* mutation carriers (Brettschneider et al., 2014). In favour of a neurodevelopmental process, the trajectory of structural and functional imaging deficits in *C9orf72* mutation carriers is deemed to be relatively similar to expected age-related changes observed in controls (Lee et al., 2016; Caverzasi et al., 2019). Some longitudinal studies do not detect progression, albeit short follow-up intervals may be ill-suited to detect subtle progressive changes (Panman et al., 2019). In addition, animal studies suggest that *C9orf72* protein plays a fundamental role in central nervous system development (Yeh et al., 2018) and have observed altered synaptic structure in *C9orf72* mutation carriers (Xu & Xu, 2018). The reality may lie somewhere in-between with pre-symptomatic radiological changes capturing both early phases of neurodegeneration superimposed on pre-existing neurodevelopmental abnormalities (Lulé et al., 2020a).

Presymptomatic radiological observations may have important practical implications: predicting phenotype, heralding phenoconversion, tracking disease progression, and optimising the timing of clinical trial enrolment. The prospect of predicting subsequent clinical phenotype is seldom addressed in the current literature. This is important to explore in longitudinal studies traversing phenoconversion as some genotypes, such as *C9orf72*, may evolve into distinctly different clinical phenotypes along the ALS-FTD spectrum (Chipika et al., 2020; Omer et al., 2017; Chipika et al., 2021). Presymptomatic spinal cord pathology in hexanucleotide expansion carriers is likely to predict ALS-FTD rather than FTD (Querin et al. 2019a) highlighting the role of quantitative cord imaging techniques (Mendili et al., 2019; Bede et al., 2012). While machine-learning (ML) frameworks have been successfully applied to imaging data of symptomatic patient cohorts (McKenna et al., 2022; Bede et al., 2021b, c), their potential has not been systematically examined in presymptomatic mutation carriers. The role of imaging in clinical trials is of particular interest given the advances in gene-specific therapeutic strategies, such as antisense oligonucleotides (Tran et al., 2022; Shing et al., 2021). The exact timing of early intervention is yet to be defined. Lessons from other neurodegenerative disorders suggest that therapeutic efficacy should be first demonstrated in early symptomatic cohorts, and later across the spectrum of disease (Tabrizi et al., 2012). Potential benefits may not be appreciated if tested in exclusively pre-symptomatic cohorts (Tabrizi et al., 2012). In genetic FTD, very early symptomatic disease may be captured by combining the accelerating peri-diagnostic radiological changes in tandem with fluid biomarkers (Swift et al., 2021); thus facilitating optimal timing for clinical trial enrolment. Imaging could also be used to track disease burden objectively in individual subjects (McKenna et al., 2021; Tahedl et al., 2021). Similarly to other neurodegenerative conditions, longitudinal imaging studies in FTD should be complemented by wet biomarkers and comprehensive clinical profiling (Blasco et al., 2018; Burke et al., 2016; Pradat et al., 2020; Querin et al., 2019b). Future clinical trials would need to adhere to standardised terminology because the terms ‘asymptomatic’, ‘pre-symptomatic’, ‘pre-symptomatic’, ‘pre-clinical’, ‘pre-manifest’ and ‘prodromal’ are used inconsistently and often interchangeably. Recently proposed nomenclature divides the overarching ‘pre-symptomatic’ phase into: ‘pre-manifest’ whereby there is only biomarker evidence of disease; and ‘prodromal’ whereby there may be detectable clinical signs without fulfilling the diagnostic criteria (Benatar et al., 2019).

While there is a likely reporting bias for significant radiological changes, pre-symptomatic changes are often not detected. The study population sometimes comprises an admixed cohort of pathogenic mutation carries, ages, subsequent clinical phenotypes, and individual modifying factors (Schuster et al., 2015). Familial FTD is a relatively low-incidence condition that sometimes leads to admixed studies of pre-symptomatic *C9orf72*, *GRN* and *MAPT* mutation carriers to boost sample sizes despite each genetic condition exhibiting relatively specific imaging signatures. However, if the participants are stratified according to the underlying genotype, studies may be underpowered to ascertain pathological changes (Feis et al., 2019). Clinical phenotypes are also associated with distinct patterns of lobar atrophy, particularly *GRN* which may evolve to bvFTD or nfvPPA phenotypes (Premi et al., 2016; Lee et al., 2019). Recent studies have shown that the pre-symptomatic cascade may be relatively uniform in nfvPPA and more diverse in bvFTD (Panman et al., 2021). The interval to phenoconversion is likely to be a key determinant of the success in detecting presymptomatic changes. Concomitant GM and WM degeneration can be often detected a few years before symptom onset (Panman et al., 2019; Borrego-Écija et al., 2021; Feis et al., 2019). The characteristic asymmetric cortical atrophy associated with *GRN* is only typically appreciated within this time window (Fumagalli et al. 2018). The inclusion of participants with considerable differences in their estimated interval to symptom onset, especially younger participants, may preclude the detection of subtle pre-symptomatic radiological changes that evolve closer to the time of symptom onset (Borrego-Écija et al., 2021; Feis et al., 2019). For example, GM degeneration may be detected in *MAPT* and *GRN* mutation carriers 2-years before symptom onset, but not in those who did not convert to during follow-up (Jiskoot et al., 2019). Differences in terminology, methodological strategies, ROI priorities, demographic profiles, choice of controls, statistical thresholds all add the apparent inconsistency of findings in the literature. Longitudinal studies are needed to capture progressive changes which are not appreciated in cross-sectional analyses (Dopper et al., 2016), but the follow-up interval may be too short to detect insidious changes and map propagation patterns (Panman et al., 2019). While imaging changes in mutation carriers offer invaluable insights into the relatively arcane presymptomatic phase of the disease, these observations may not be transferable to sporadic FTD.

## Conclusions

Genotype-specific imaging changes may be detected several years before symptom onset in pre-symptomatic familial FTD mutation carriers, but robust multimodal, multi-timepoint longitudinal studies are required for the nuanced characterisation of the evolution of structural and functional changes.

## Supplementary Information

Below is the link to the electronic supplementary material.

Supplementary file1 (DOCX 342 KB)

## Data Availability

Not applicable.

## Glossary

1H-MRS: Proton MR spectroscopy
AD: Axial diffusivity
AD: Alzheimer's disease
ALS: Amyotrophic lateral sclerosis
AP: Anteroposterior
ASL: Arterial spin labelling
BG: Basal ganglia
BPF: Brain parenchymal fractions
C9orf72: Chromosome 9 open reading frame 72
CC: Corpus callousm
Cho: Choline
Cr: Creatine
CR: Corona radiata
CST: Corticospinal tract
CNS: Central nervous system
DKI: Diffusion kurtosis imaging
DAT: Dopamine active transporter scan
DTI: Diffusion tensor imaging
EPI: Echo-planar imaging
FA: Fractional anisotropy
FDG-PET: Fluorodeoxyglucose-positron emission tomography
FLAIR: Fluid-attenuated inversion recovery
fMRI: Functional MRI
FOV: Field of view
FTD: Frontotemporal dementia
GABA: γ-aminobutyric acid
Gln: Glutamine
Glu: Glutamate
GM: Grey matter
GRN: Progranulin
HARDI: High angular resolution diffusion imaging
HC: Healthy control
HD: Huntington's disease
Lt: Left
MD: Mean diffusivity
MAPT: Microtubule-associated protein tau
mI: Myoinositol
ML: Machine-learning
MND: Motor neuron disease
MRI: Magnetic resonance imaging
MRS: Magnetic resonance spectroscopy
NAA: N-acetyl-aspartate
NODDI: Neurite orientation dispersion and density imaging
PET: Positron emission tomography
PLIC: Posterior limb of the internal capsule
PLS: Primary lateral sclerosis
PMC: Primary motor cortex
PRISMA: Preferred reporting items for systematic reviews and meta-analyses
QSM: Quantitative susceptibility mapping
rCBF: Regional cerebral blood flow
RD: Radial diffusivity
ROI: Region of interest
rs-fMRI: Resting-state functional MRI
Rt: Right
SPECT: Single-photon emission computerized tomography
SWI: Susceptibility weighted imaging
T: Tesla
T1w: T1-weighted imaging
TBSS: Tract-based spatial Statistics
TIV: Total intracranial volume
UMN: Upper motor neuron
VBM: Voxel-based morphometry
WM: White matter
WMH: White matte hyperintensity
