# Supplementary material for: Pre-symptomatic radiological changes in frontotemporal dementia: propagation characteristics, predictive value and implications for clinical trials
Source: Brain Imaging Behav. 2022 Aug 3;16(6):2755–67. doi: 10.1007/s11682-022-00711-z (PMC9712335; doi:10.1007/s11682-022-00711-z)
Supplement: Supplementary file 1 — Supplementary file1 (DOCX 342 KB) [file 11682_2022_711_MOESM1_ESM.docx]

**Supplementary Table 1.** Imaging studies of pre-symptomatic *C9orf72* mutation carriers

| **First author, year of publication** | **Study groups and cohort sizes** | **Study design** | **Follow-up** | **Imaging methods** | | **Key findings in pre-symptomatic cohorts** |
| --- | --- | --- | --- | --- | --- | --- |
| **Structural MRI** | | | | | | |
| Bertrand et al, 2018 (1) | Pre-symptomatic C9orf72 n= 41  Controls n=39 | Cross-sectional  Case control | N/A | MRI – TIV and ROI analysis, DTI | | Pre-symptomatic C9orf72: frontal, inferior temporal, parietal and right thalamus atrophy; and WM alterations in CST and frontotemporal tracts. Less extensive changes in mutation carriers aged <40 years |
| Bocchetta et al, 2021(2) | Pre-symptomatic MAPT n=47  Symptomatic MAPT n=20  Pre-symptomatic GRN n=125  Symptomatic GRN n=43  Pre-symptomatic C9orf72 n=107  Symptomatic C9orf72 n=63  Controls n=298 | Cross-sectional  Case control | N/A | MRI – Cortical and subcortical volumes | | Pre-symptomatic and mild-symptomatic C9orf72: reduced cortical volume of the insula, dorsolateral prefrontal, motor, dorsolateral temporal, lateral parietal and occipital cortex.  Pre-symptomatic C9orf72: reduced subcortical volume of the thalamus (pulvinar, lateral geniculate nucleus, and lentiform nucleus), basal ganglia (putamen) and medial temporal regions (hypothalamus, amygdala), reduced cerebellar volume (lobules VIIa-Crus II and VIIb). |
| Cash et al, 2018(3) | Pre-symptomatic GRN n=65  Symptomatic GRN n=12  Pre-symptomatic C9orf72 n=40  Symptomatic C9orf72 n=25  Pre-symptomatic MAPT n=23  Symptomatic MAPT n=10  Controls n=144 | Cross-sectional  Case control | N/A | MRI - VBM | | Pre-symptomatic mutation carriers: significant decrease in GM volume in the insula.  Pre-symptomatic C9orf72: loss of GM volume in the thalamus, superior and medial temporal, inferior frontal, inferior parietal and right superior posterior cerebellum (Crus I) depending on statistical threshold. |
| Caverzasi et al, 2019 (4) | Pre-symptomatic C9orf72 n=15  Controls n=67 | Cross-sectional  Case control | N/A | MRI – cortical thickness and local gyrification index | | Pre-symptomatic C9orf72: abnormal low gyrification in left frontal and right parieto-occipital regions decades before symptom onset. These areas showed no corresponding cortical thickness abnormality. |
| Convery et al, 2020(5) | Pre-symptomatic C9orf72 n=73  Symptomatic C9orf72 n=31  Pre-symptomatic GRN n=104  Symptomatic GRN n=24  Pre-symptomatic MAPT n=39  Symptomatic MAPT n=10  Controls n=181 | Cross-sectional  Case control | N/A | MRI - VBM | | Pre-symptomatic C9orf72: no difference in pain perception |
| Cury et al, 2019(6) | Pre-symptomatic GRN n=53  Pre-symptomatic C9orf72 n=34  Pre-symptomatic MAPT n=26  Controls n= 98 | Cross-sectional  Case control | N/A | MRI – large diffeomorphic deformation metric mapping | | Pre-symptomatic mutation carriers: altered shape of anterior thalamus at least 5 years before expected symptom onset. |
| Feis et al, 2018(7) | Pre-symptomatic GRN n=35;  Pre-symptomatic C9orf72 n=72  Pre-symptomatic MAPT n=8;  Controls n = 48 | Cross-sectional  Case control | N/A | MRI – GM and WM density,DTI  rs-fMRI | | Pre-symptomatic mutation carriers: best performing multimodal classification model differentiated from controls using only WM features (radial diffusivity and WM density) suggesting that earliest FTD-related changes occur in WM. |
| Floeter et al, 2016 (8) | Pre-symptomatic C9orf72 n= 7  Symptomatic C9orf72 n=20  Sporadic ALS n=22  Controls n=28 | Longitudinal  Case-control | 6-18 months | MRI – brain volumes and cortical thickness analyses | | Pre-symptomatic C9orf72: no difference at cross-sectional or longitudinal analyses |
| Fumagalli et al, 2018(9) | Pre-symptomatic GRN n= 66  Symptomatic GRN n=17  Pre-symptomatic C9orf72 n=42  Symptomatic C9orf72 n=31  Pre-symptomatic MAPT n=24  Symptomatic MAPT n=15  Controls n=148 | Cross-sectional  Case control | N/A | MRI- VBM | | Pre-symptomatic C9orf72: visual rating scales could not identify GM atrophy |
| Gazzina et al, 2019(10) | Pre-symptomatic GRN n=65  Pre-symptomatic C9orf72 n=31  Pre-symptomatic MAPT n=20  Controls n=113 | Longitudinal  Case-control | 4 years | MRI – GM volume | | Highly educated pre-symptomatic mutation carriers: greater brain maintenance with slower GM volume loss. |
| Le Blanc et al, 2020 (11) | Pre-symptomatic C9orf72 n=83  Symptomatic C9orf72 n= 54  Control n=249 | Cross-sectional  Case control | N/A | MRI – cortical thickness, and cortical surface area | | Pre-symptomatic C9orf72: reduced cortical thickness and surface area in the medial frontoparietal lobes and some frontal, parietal and temporal regions; faster rate of cortical thinning and surface area loss in a similar anatomical distribution to involvement in symptomatic subjects. |
| Lulé et al, 2020(12) | Pre-symptomatic C9orf72 n=21  Pre-symptomatic SOD1 n=15  Controls n=91 | Longitudinal  Case-control | 12-months | MRI - DTI | | Pre-symptomatic C9orf72: loss of WM integrity in inferior and orbitofrontal cortical areas. No progression over 1-year |
| Malpetti et al, 2021(13) | Pre-symptomatic GRN n=142  Pre-symptomatic C9orf72 n=108  Pre-symptomatic MAPT n=54  Controls n=296 | Longitudinal  Case-control | 2-years | MRI – GM volume | | Pre-symptomatic mutation carriers: apathy progression was associated with low GM volume in frontal and cingulate. |
| Olney et al, 2020(14) | Pre-symptomatic mutation carriers n=103  Mild symptomatic carriers n=43  Dementia n=72  Controls n=102 | Cross-sectional  Case control | N/A | MRI – cortical volumes | | Pre-symptomatic mutation carriers: reduced frontal and temporal volumes.  Pre-symptomatic C9orf72: early right thalamus and left peri-insular involvement. |
| Panman et al, 2019(15) | Pre-symptomatic GRN n=33  Pre-symptomatic MAPT n=15  Pre-symptomatic C9orf72 n=12  Controls n=53 | Longitudinal  Case-control | 2-years | MRI – VBM, cortical thickness. TBSS | | Pre-symptomatic C9orf72: reduced GM volume in the insula and cerebellum; and altered WM integrity in the anterior thalamic radiation at baseline and follow-up. These cross-sectional changes remained stable on interval follow-up. |
| Papma et al, 2017 (16) | Pre-symptomatic C9orf72 n=18  Control n=15 | Cross-sectional  Case control | N/A | MRI – VBM and DTI | | Pre-symptomatic C9orf72: loss of integrity of the frontal WM tracts and thalamic radiation. In a subgroup, of those aged >40 years, there was also loss of GM volume in the thalamus, cerebellum, parietal and temporal cortex. |
| Popuri et al, 2018(17) | Pre-symptomatic C9orf72 n=15  Pre-symptomatic GRN n= 9  Controls n=38 | Cross-sectional  Case control | N/A | MRI – cortical thickness and subcortical GM volumes | | Pre-symptomatic C9orf72: cortical thinning in the temporal, parietal and frontal regions; reduced volumes in the bilateral thalamus and left caudate. |
| Premi et al, 2017(18) | Pre-symptomatic GRN n=61  Pre-symptomatic MAPT n=14  Pre-symptomatic C9orf72 n=33  Controls n=123 | Cross-sectional  Case control | N/A | MRI – cortical, subcortical and cerebellar volumes | | Pre-symptomatic mutation carriers: education level directly affects GM volume;  TMEM106B genotype enhances the benefit of cognitive reserve on brain structure. |
| Querin et al, 2019 (19) | Pre-symptomatic C9orf72 n= 40  Controls n=32 | Longitudinal  Case-control | 18-months | MRI – total, GM and WM cervical spinal cord cross-sectional area, DTI | | Pre-symptomatic C9orf72 aged >40 years: baseline - cervical spinal cord WM atrophy; follow-up - reduced FA in CST  Pre-symptomatic C9orf72 aged >40 years with a family history of ALS: baseline - also reduced FA in CST |
| Rohrer et al, 2015(20) | Pre-symptomatic C9orf72 n=18  Symptomatic C9orf72 n=16  Pre-symptomatic MAPT n=15  Symptomatic MAPT n=11  Pre-symptomatic GRN n=45  Symptomatic GRN n=13  Controls n=102 | Cross-sectional  Case control | N/A | MRI – cortical and subcortical volumes | | Pre-symptomatic C9orf72: thalamus, insula and posterior cortical involvement 25 years before expected onset; frontal and temporal lobes at 20 years before expected onset, and cerebellum 10-years before symptom onset. |
| Russell et al, 2020(21) | Pre-symptomatic GRN n= 123  Symptomatic GRN n= 32  Pre-symptomatic C9orf72 n= 106  Symptomatic C9orf72 n= 53  Pre-symptomatic MAPT n= 49  Symptomatic MAPT n= 18 | Cross-sectional | N/A | MRI - VBM | | Late pre-symptomatic C9orf72: performed lower in social cognition tests compared to controls  Pre-symptomatic and symptomatic C9orf72 group: these tests correlated with involvement of insula, left frontal lobe, left basal ganglia, right amygdala, and right temporal lobe. |
| Staffaroni et al 2021(22) | Pre-symptomatic mutation carriers n=46  Symptomatic mutation carriers n=81  Controls n=101  Reference n=383 | Longitudinal | 2-years | MRI – GM volume | | Pre-symptomatic mutation carriers: individualized atrophy scores predict phenoconversion, achieving 90% accuracy |
| Sudre et al, 2017(23) | Pre-symptomatic C9orf72 n= 28  Symptomatic C9orf72 n= 23  Pre-symptomatic MAPT n= 8  Symptomatic MAPT n= 13  Pre-symptomatic GRN n= 25  Symptomatic GRN n= 7  Controls n= 76 | Cross-sectional  Case control | N/A | MRI – automatic segmentation of WMH using T1 and T2 weighted images | | Pre-symptomatic or symptomatic C9orf72: no significant differences in WMH |
| Tavares et al, 2019(24) | Pre-symptomatic GRN n= 29  Pre-symptomatic C9orf72 n= 13  Pre-symptomatic MAPT n= 4  Controls n= 56 | Longitudinal  Case-control | 1-year | MRI – ventricular volumes | | Pre-symptomatic FTD: ventricular volume difference begins on average 4 years before expected symptom onset. |
| Walhout et al, 2015 (25) | Pre-symptomatic C9orf72 n=16  Symptomatic C9orf72 n=14  Control n=51 | Cross-sectional  Case control | N/A | MRI – cortical thickness, subcortical volumes and DTI | | Pre-symptomatic C9orf72: thinned temporal, parietal, occipital cortices and smaller left caudate and putamen.  The involvement of the primary motor regions and motor-related tracts were found exclusively in patients with ALS. |
| Wen et al, 2019(26) | Pre-symptomatic C9orf72 n=38  Control n=29 | Cross-sectional  Case control | N/A | MRI – volumetry, DTI, NODDI with ROI analysis | | Pre-symptomatic C9orf72: NODDI had greater sensitivity than DTI at detecting CST and frontotemporal WM tracts abnormalities. It also detected more cortical abnormalities in the frontal, temporal parietal, occipital and insular regions. |
| **Functional MRI** | | | | | | |
| Feis et al, 2019(27) | Pre-symptomatic GRN n=35  Pre-symptomatic MAPT n=8  Pre-symptomatic C9orf72 n=12  Controls = 48 | Longitudinal  Case-control | 6-years | MRI-GMD, DTI  rs-fMRI | | Pre-symptomatic mutation carriers: MRI classification scores remain similar to controls until close to symptom onset. |
| Lee et al, 2017(28) | Pre-symptomatic C9orf72 n=15  Control n=15 | Cross-sectional  Case control | N/A | MRI – rs-fMRI, VBM, DTI | | Pre-symptomatic C9orf72: GM (cingulate, insula, thalamus, striatum)’ WM (corpus callosum, cingulum bundles, CST, uncinate fasciculus and inferior longitudinal fasciculus); and connectivity deficits (most prominent in salience and medial pulvinar thalamus networks) as early as 4^th^ decade of life. |
| Mutsaerts et al, 2019(29) | Pre-symptomatic C9orf72 n= 34  Pre-symptomatic GRN n= 55  Pre-symptomatic MAPT n= 18  Controls n= 113 | Cross-sectional  Case control | N/A | MRI – Arterial spin labelling | | Pre-symptomatic mutation carriers: lower blood flow in FTD regions up to 12.5 years before expected symptom onset. |
| Premi et al, 2019 (30) | Pre-symptomatic C9orf72 n= 82  Pre-symptomatic GRN n= 122  Pre-symptomatic MAPT n= 45  Controls n= 223 | Cross-sectional  Case control | N/A | rs-fMRI | | Pre-symptomatic C9orf72: reduced meta-states numbers and reduced meta-state changes.  Pre-symptomatic mutation carriers: altered connectivity with diminished dynamic fluidity and restricted dynamic range. |
| Rittman et al, 2019(31) | Pre-symptomatic C9orf72 n= 17  Symptomatic C9orf72 n= 12  Pre-symptomatic GRN n= 40  Symptomatic GRN n= 6  Pre-symptomatic MAPT n= 13  Symptomatic MAPT n= 11  Controls n= 86 | Cross-sectional  Case control | N/A | Task-free fMRI | | Pre-symptomatic mutation carriers: preserved efficient topological organisation of the brains functional network in the years leading up to expected symptom onset, despite pre-symptomatic loss of brain volume and functional connections. |
| Shoukry et al, 2020 (32) | Pre-symptomatic C9orf72 n=15  Symptomatic C9orf72 n=27  Controls n=48 | Longitudinal  Case-control | 6-months  18-months | MRI – rs-fMRI | | Pre-symptomatic C9orf72: stable reduced functional connectivity in the thalamic networks; and gradually progressive reduced functional connectivity in frontal, temporal and motor networks in a pattern similar to symptomatic carriers. |
| Tsvetanov et al, 2021(33) | Pre-symptomatic C9orf72 n= 39  Pre-symptomatic GRN n= 63  Pre-symptomatic MAPT n=19  Controls n=134 | Cross-sectional  Case control | N/A | MRI – GM volume  Rs-fMRI | | Pre-symptomatic mutation carriers: maintenance of functional network connectivity may enable maintenance of cognitive performance in the presence of progressive brain atrophy for years before the onset of symptoms. |
| Waugh et al, 2021 (34) | Pre-symptomatic C9orf72 n = 15  Symptomatic C9orf72 n=27  Controls n=34 | Longitudinal  Case-control | 18-months | MRI – rs-fMRI, DTI, cortical thickness and volume analyses | | Pre-symptomatic C9orf72: increased network homogeneity adjacent to the areas with decreased functional connectivity in symptomatic C9orf72. The functional connectivity trajectory differs from controls and symptomatic C9orf72;  No difference in cortical volumes or cortical thickness or DTI metrics |
| **Positron Emission Tomography** | | | | | | |
| De Vocht et al, 2020 (35) | Pre-symptomatic C9orf72 n = 17  Controls n=25 | Cross-sectional  Case-Control | N/A | | [18F] FDG-PET | Pre-symptomatic C9orf72: relative hypometabolism in the insular cortex, central opercular cortex, basal ganglia and thalami; and relative hypermetabolism in the pre-central gyrus and precuneus cortex. |
| Malpetti et al, 2021(36) | Pre-symptomatic C9orf72 n = 3  Symptomatic C9orf72 n=1  Controls n= 19 | Cross-sectional  Case-Control | N/A | | MRI and [^11^ C]UCB-J PET | Pre-symptomatic C9orf72: loss of synaptic density in the thalamus, most marked in the pulvinar and ventral-posterior thalamic subregion compared to controls. |
| Popuri et al, 2021 (37) | Pre-symptomatic C9orf72 n= 15  Controls n=20 | Cross-sectional  Case-Control | N/A | | MRI and [18F] FDG-PET | Pre-symptomatic C9orf72: regional hypometabolism detected involving the cingulate gyrus, frontal and temporal cortices (L>R) and bilateral thalami before GM changes and up to 10-years before symptom onset. |

**Supplementary Table 2**. Imaging studies of pre-symptomatic GRN mutation carriers

| **First author, year of publication** | **Study groups and cohort sizes** | **Study design** | **Follow-up** | **Imaging methods** | **Key findings in pre-symptomatic cohorts** |
| --- | --- | --- | --- | --- | --- |
| **Structural MRI** | | | | | |
| Bocchetta et al, 2021(2) | Pre-symptomatic MAPT n=47  Mild symptomatic MAPT n-13  Symptomatic MAPT n=20  Pre-symptomatic GRN n=125  Mild symptomatic GRN n=30  Symptomatic GRN n=43  Pre-symptomatic C9orf72 n=107  Mild symptomatic C9orf72 n=32  Symptomatic C9orf72 n=63  Controls n=298 | Cross-sectional  Case control | N/A | MRI – Cortical and subcortical volumes | Pre-symptomatic GRN: no difference in cortical or subcortical volume compared to controls. |
| Borrego-Écija et al, 2021(38) | Pre-symptomatic GRN n=100  Controls n=94 | Cross-sectional  Case control | N/A | MRI – cortical thickness | Pre-symptomatic GRN: age-related frontal cortical thinning, especially left superior frontal cortex.  No difference in cortical thickness compared with control. |
| Borroni et al, 2008 (39) | Pre-symptomatic GRN n=7  Controls n=15 | Cross-sectional  Case control | N/A | MRI – VBM and DTI | Pre-symptomatic GRN: no difference in GM and WM volume compared to controls;  DTI -reduced FA in left uncinate fasciculus and left inferior occipitofrontal fasciculus. |
| Cash et al, 2018(3) | Pre-symptomatic GRN n=65  Symptomatic GRN n=12  Pre-symptomatic C9orf72 n=40  Symptomatic C9orf72 n=25  Pre-symptomatic MAPT n=23  Symptomatic MAPT n=10  Controls n=144 | Cross-sectional  Case control | N/A | MRI - VBM | Pre-symptomatic mutation carriers: GM volume loss involving the insula.  Pre-symptomatic GRN mutation carriers: striatal, posterior frontal, anterior temporal, and parietal lobe atrophy. |
| Chen et al, 2020(40) | Pre-symptomatic GRN n=8  Symptomatic GRN n=5  Controls n=10 | Longitudinal  Case-control | Median 3 years (1.0-9.8) | MRI – TBM-SyN | Asymptomatic GRN: higher annual rates of frontal and parietal lobe cortical atrophy compared to controls |
| Convery et al, 2020(5) | Pre-symptomatic GRN n=104  Symptomatic GRN n=24  Pre-symptomatic C9orf72 n=73  Symptomatic C9orf72 n=31  Pre-symptomatic MAPT n=39  Symptomatic MAPT n=10  Controls n=181 | Cross-sectional  Case control | N/A | MRI - VBM | Pre-symptomatic and symptomatic GRN: no difference in pain perception compared with controls. |
| Cury et al, 2019(6) | Pre-symptomatic GRN n=53  Pre-symptomatic C9orf72 n=34  Pre-symptomatic MAPT n=26  Controls n= 98 | Cross-sectional  Case control | N/A | MRI – large diffeomorphic deformation metric mapping | Pre-symptomatic mutation carriers: altered shape of anterior thalamus at least 5 years before expected symptom onset. |
| Fumagalli et al, 2018(9) | Pre-symptomatic GRN n= 66  Symptomatic GRN n=17  Pre-symptomatic C9orf72 n=42  Symptomatic C9orf72 n=31  Pre-symptomatic MAPT n=24  Symptomatic MAPT n=15  Controls n=148 | Cross-sectional  Case control | N/A | MRI- VBM | Pre-symptomatic GRN carriers: visual rating scales could not identify GM atrophy. |
| Gazzina et al, 2018(41) | Pre-symptomatic GRN n=19  Controls n=17 | Cross-sectional  Case control | N/A | MRI – cortical volume, thickness and surface area | Pre-symptomatic GRN: increased cortical thickness in the supramarginal and superior parietal gyri; decreased surface area in the precuneus and inferior parietal gyrus; no significant volume changes. Transcranial magnetic stimulation parameters of intracortical inhibition and facilitation correlated with right insular reduced surface area and volume. |
| Gazzina et al, 2019(10) | Pre-symptomatic GRN n=65  Pre-symptomatic C9orf72 n=31  Pre-symptomatic MAPT n=20  Controls n=113 | Longitudinal  Case-control | 4 years | MRI – GM volume | Highly educated pre-symptomatic mutation carriers: greater brain maintenance with slower GM volume loss. |
| Jiskoot et al, 2019(42) | Pre-symptomatic GRN n=30  Pre-symptomatic MAPT n=13  Controls n=30 | Longitudinal  Case-control | 4 years | MRI – VBM, TBSS, DTI, ROI analysis | Pre-symptomatic GRN: greater loss of WM integrity involving the genu of the corpus callosum in the 2-years approaching phenoconversion compared to MAPT mutation carriers. |
| Malpetti et al, 2021(13) | Pre-symptomatic GRN n=142  Pre-symptomatic C9orf72 n=108  Pre-symptomatic MAPT n=54  Controls n=296 | Longitudinal  Case-control | 2-years | MRI – GM volume | Pre-symptomatic carriers: apathy progression associated with baseline low GM volume in frontal and cingulate regions. |
| Milanesi et al, 2013 (43) | Pre-symptomatic GRN n=14  Symptomatic GRN n=15  FTD GRN negative n=16  Controls n=11 | Cross-sectional  Case control | N/A | MRI - VBM | Pre-symptomatic and symptomatic GRN: higher levels of TMEM40 gene expression were associated with greater parietal atrophy; and higher LY6G6F were associated with greater superior frontal gyrus atrophy. |
| Olm et al, 2018 (44) | Pre-symptomatic GRN n=11  Controls n=11 | Longitudinal  Case-control | Mean 2.2 years | MRI – GM density, DWI | Pre-symptomatic GRN: greater annualised GMD changes in right orbitofrontal and left occipital cortices; FA change in the right superior longitudinal fasciculus and frontal corpus callosum. |
| Olney et al, 2020(14) | Pre-symptomatic mutation carriers n=103  Mild symptomatic carriers n=43  Dementia n=72  Controls n=102 | Cross-sectional  Case control | N/A | MRI – cortical volumes | Pre-symptomatic mutation carriers: reduced frontal and temporal volumes compared to controls.  Pre-symptomatic GRN: early posterior temporal and parietal region involvement |
| Panman et al, 2019 (15) | Pre-symptomatic GRN n=33  Pre-symptomatic MAPT n=15  Pre-symptomatic C9orf72 n=12  Controls n=53 | Longitudinal  Case-control | 2-years | MRI – VBM, cortical thickness. TBSS | Pre-symptomatic GRN: no significant cross-sectional or longitudinal differences detected in GM or WM |
| Panman et al, 2021 (45) | Pre-symptomatic GRN n=56  Symptomatic GRN n=35  Controls n=35 | Cross-sectional | N/A | MRI – Volume and DTI FA | Pre-symptomatic GRN: WM tracts were affected before GM volume. Left hemisphere was affected before the right hemisphere. The biomarker cascade in bvFTD showed more uncertainty than nfvPPA phenotype. |
| Paternicò et al, 2016(46) | Pre-symptomatic GRN n=11  Controls n=11  Symptomatic GRN n=14  FTD GRN negative n=28  Controls n=15 | Cross-sectional  Case control | N/A | MRI - segmentation of WMH using T1 and T2 weighted images | Pre-symptomatic GRN: no significant difference in WMH burden compared to controls |
| Popuri et al, 2018(17) | Pre-symptomatic C9orf72 n=15  Pre-symptomatic GRN n= 9  Controls n=38 | Cross-sectional  Case control | N/A | MRI – cortical thickness and subcortical GM volumes | Pre-symptomatic GRN: no difference in cortical thickness or subcortical volumes compared with controls. |
| Premi et al, 2017(18) | Pre-symptomatic GRN n=61  Pre-symptomatic MAPT n=14  Pre-symptomatic C9orf72 n=33  Controls n=123 | Cross-sectional  Case control | N/A | MRI – cortical, subcortical and cerebellar volumes | Pre-symptomatic mutation carriers: education level directly affects GM volume;  TMEM106B genotype enhances the benefit of cognitive reserve on brain structure. |
| Rohrer et al, 2015(20) | Pre-symptomatic C9orf72 n=18  Symptomatic C9orf72 n=16  Pre-symptomatic MAPT n=15  Symptomatic MAPT n=11  Pre-symptomatic GRN n=45  Symptomatic GRN n=13  Controls n=102 | Cross-sectional  Case control | N/A | MRI – cortical and subcortical volumes | Pre-symptomatic GRN: insula involvement at 15-years before expected onset; temporal and parietal involvement at 10-years before expected onset; striatum involvement and cortical asymmetry at 5-years before expected onset; |
| Russell et al, 2020(21) | Pre-symptomatic GRN n= 123  Symptomatic GRN n= 32  Pre-symptomatic C9orf72 n= 106  Symptomatic C9orf72 n= 53  Pre-symptomatic MAPT n= 49  Symptomatic MAPT n= 18 | Cross-sectional | N/A | MRI - VBM | Pre-symptomatic GRN: no different than controls in social cognition tests.  Pre-symptomatic and symptomatic GRN: these tests correlated with left hemisphere predominant network involvement of insula, frontal lobe, inferomedial temporal lobe, cingulate, basal ganglia and thalamus. |
| Staffaroni et al 2021(22) | Pre-symptomatic mutation carriers n=46  Symptomatic mutation carriers n=81  Controls n=101  Reference n=383 | Longitudinal | 2-years | MRI – GM volume | Pre-symptomatic mutation carriers: individualized atrophy scores predict phenoconversion, achieving 90% accuracy |
| Sudre et al, 2017(23) | Pre-symptomatic GRN n= 25  Symptomatic GRN n= 7  Pre-symptomatic C9orf72 n= 28  Symptomatic C9orf72 n= 23  Pre-symptomatic MAPT n= 8  Symptomatic MAPT n= 13  Controls n= 76 | Cross-sectional  Case control | N/A | MRI – automatic segmentation of WMH using T1 and T2 weighted images | Pre-symptomatic GRN: no differences in WMH; associated increased WMH volume expected years from onset. |
| Sudre et al, 2019(47) | Pre-symptomatic GRN n=101  (Longitudinal n=39)  Symptomatic GRN n=32  (Longitudinal n=12)  Controls n=203  (Longitudinal n=73) | Longitudinal  Case-control | Not specified  Annual MRI imaging | MRI – automatic segmentation of WMH and GM using T1 and T2 weighted images | Pre-symptomatic GRN: WMH load greater in parietal and occipital regions, with a trend towards periventricular location compared to controls; no difference in longitudinal increase in WMH burden; WMH associated with increased GM volume loss, executive dysfunction, and markers of WM damage (neurofilament light chain, glial fibrillary acidic protein). |
| Tavares et al, 2019(24) | Pre-symptomatic C9orf72 n= 13  Pre-symptomatic GRN n= 29  Pre-symptomatic MAPT n= 4  Controls n= 56 | Longitudinal  Case-control | 1-year | MRI – ventricular volumes | Pre-symptomatic FTD: ventricular volume difference begins on average 4 years before expected symptom onset. |
| **Functional MRI** | | | | | |
| Borroni et al, 2012 (48) | Pre-symptomatic GRN n=9  Symptomatic GRN n=7  FTD GRN negative n=16  Controls n=24 | Cross-sectional  Case control | N/A | MRI - VBM  rs-fMRI | Pre-symptomatic GRN: no GM atrophy; increased connectivity in salience network; no changes in default mode network |
| Dopper et al, 2014 (49) | Pre-symptomatic GRN n=28  Pre-symptomatic MAPT n=9  Controls n=38 | Cross-sectional  Case control | N/A | MRI – VBM, TBSS  rs-fMRI | Pre-symptomatic mutation carriers: no difference in GM volume  Pre-symptomatic GRN carriers: decreased FA in the right anterior and superior corona radiata, anterior thalamic radiation, superior and inferior longitudinal fasciculus, inferior fronto-occipital fasciculus, forceps minor and anterior and posterior limb of internal capsule; decreased functional connectivity of the anterior midcingulate cortex. |
| Dopper et al, 2016 (50) | Pre-symptomatic MAPT n = 11  Pre-symptomatic GRN n = 23  Controls n=31 | Longitudinal  Case-control | Mean 2.2 (2.1-2.3) years | MRI – Arterial spin labelling | Pre-symptomatic GRN carriers: frontoparietal hypoperfusion |
| Feis et al, 2018(7) | Pre-symptomatic GRN n=35;  Pre-symptomatic C9orf72 n=72  Pre-symptomatic MAPT n=8;  Controls n = 48 | Cross-sectional  Case control | N/A | MRI – GM and WM density, DTI  rs-fMRI | Pre-symptomatic mutation carriers: best performing multimodal classification model differentiated from controls using only WM features (radial diffusivity and WM density) suggesting that earliest FTD-related changes occur in WM. |
| Feis et al, 2019(51) | Pre-symptomatic GRN n=28  Pre-symptomatic MAPT n=11  Controls = 36 | Cross-sectional  Case control | N/A | MRI – VBM, DTI, TBSS  rs-fMRI | Pre-symptomatic mutation carriers: no difference in GM volume, DTI metrics or functional connectivity. |
| Feis et al, 2019(27) | Pre-symptomatic GRN n=35  Pre-symptomatic MAPT n=8  Pre-symptomatic C9orf72 n=12  Controls = 48 | Longitudinal  Case-control | 6-years | MRI-GMD, DTI  rs-fMRI | Pre-symptomatic mutation carriers: MRI classification scores similar to controls based until close to symptom onset. |
| Lee et al, 2019(52) | Pre-symptomatic GRN n=14  Pre-clinical GRN n=3  Controls n=30 | Cross-sectional  Case control | N/A | MRI - VBM  Task-free fMRI | Pre-symptomatic and pre-clinical GRN: widespread hyperconnectivity in the salience network, nfvPPA network, CBS network and default mode network. In particular, thalamo-cortical network hyperconnectivity is the unifying feature. |
| Mutsaerts et al, 2019(29) | Pre-symptomatic C9orf72 n= 34  Pre-symptomatic GRN n= 55  Pre-symptomatic MAPT n= 18  Controls n= 113 | Cross-sectional  Case control | N/A | MRI – Arterial spin labelling | Pre-symptomatic mutation carriers: lower blood flow in FTD regions up to 12.5 years before expected symptom onset  Pre-symptomatic GRN: bilateral anterior cingulate/paracingulate, right anterior insula/orbitofrontal, and right supramarginal/angular gyri. |
| Pievani et al, 2014(53) | Pre-symptomatic GRN n=5  Controls n=5 | Cross-sectional  Case control | N/A | MRI – cortical thickness, TBSS  rs-fMRI | Pre-symptomatic GRN: cortical thinning in the right pre-central, orbitofrontal gyrus, left rostral middle frontal gyrus; increased axial diffusivity in the right cingulum, superior longitudinal fasciculus and corticospinal tracts;  no differences in resting-state network connectivity |
| Premi et al, 2013 (54) | Pre-symptomatic GRN n= 17  Symptomatic GRN n= 12  FTD GRN negative n=20 | Cross-sectional  Case control | N/A | rs-fMRI | Pre-symptomatic GRN: cognitive reserve associated with reduced salience network connectivity. |
| Premi et al, 2014 (55) | Pre-symptomatic GRN n= 17  Symptomatic GRN n= 14  FTD GRN negative n=38 | Cross-sectional  Case control | N/A | MRI – GM volume analyses  rs-fMRI | Pre-symptomatic GRN: selective reduced regional homogeneity in left parietal regions and increased regional homogeneity in the frontal regions. |
| Premi et al, 2016 (56) | Pre-symptomatic GRN n= 17  Symptomatic GRN n= 14  Controls n=33 | Cross-sectional  Case control | N/A | MRI – VBM  rs-fMRI | Pre-symptomatic GRN: no structural alterations; local connectivity measure fractional amplitude of low frequency fluctuations (fALFF) was reduced in parietal regions and increased in prefrontal; and achieved the best classification performance from controls (accuracy 84.8%; sensitivity 76% and specificity 52%). |
| Premi et al, 2019 (30) | Pre-symptomatic C9orf72 n= 82  Pre-symptomatic GRN n= 122  Pre-symptomatic MAPT n= 45  Controls n= 223 | Cross-sectional  Case control | N/A | rs-fMRI | Pre-symptomatic GRN: no significant difference in functional connectivity compared to controls.  Pre-symptomatic mutation carriers: altered connectivity with diminished dynamic fluidity and restricted dynamic range. |
| Premi et al, 2021 (57) | Pre-symptomatic GRN n=141  Controls n=282 | Cross-sectional  Case-control |  | rs-fMRI and TIV of insula | Pre-symptomatic GRN: complex dynamic reorganisation of brain connectivity;  increased time spent in the spatial states characterised by greater activation of the insula and parietal cortices |
| Rittman et al, 2019(31) | Pre-symptomatic C9orf72 n= 17  Symptomatic C9orf72 n= 12  Pre-symptomatic GRN n= 40  Symptomatic GRN n= 6  Pre-symptomatic MAPT n= 13  Symptomatic MAPT n= 11  Controls n= 86 | Cross-sectional  Case control | N/A | Task-free fMRI | Pre-symptomatic mutation carriers: preserved efficient topological organisation of the brains functional network in the years leading up to expected symptom onset, despite pre-symptomatic loss of brain volume and functional connections. |
| Tsvetanov et al, 2021(33) | Pre-symptomatic C9orf72 n= 39  Pre-symptomatic GRN n= 63  Pre-symptomatic MAPT n=19  Controls n=134 | Cross-sectional  Case control | N/A | MRI – GM volume  Rs-fMRI | Pre-symptomatic mutation carriers: maintenance of functional network connectivity may enable maintenance of cognitive performance in the presence of progressive brain atrophy for years before the onset of symptoms. |
| **Positron Emission Tomography** | | | | | |
| Caroppo et al, 2015 (58) | Pre-symptomatic GRN n = 16  Controls n=17 | Longitudinal  Case-control | 20-months | MRI – cortical thickness  [18F] FDG-PET | Asymptomatic GRN: left lateral temporal lobe hypometabolism without atrophy; interval imaging shows progressive lateral temporal and frontal hypometabolism associated with reduced cortical thickness in the left lateral temporal lobe. |
| Jacova et al, 2013 (59) | Pre-symptomatic GRN n = 9  Controls n=11 | Cross-sectional  Case control | N/A | MRI  [18F] FDG-PET | Pre-symptomatic GRN: right anterior cerebral hypometabolism years before symptom onset, involving right medial and ventral frontal and insular cortex |

**Supplementary Table 3**. Imaging studies of pre-symptomatic MAPT mutation carriers

| **First author, year of publication** | **Study groups and cohort sizes** | **Study design** | **Follow-up** | **Imaging methods** | **Key findings in pre-symptomatic cohorts** |
| --- | --- | --- | --- | --- | --- |
| **Structural MRI** | | | | | |
| Bocchetta et al, 2021(2) | Pre-symptomatic MAPT n=47  Mild symptomatic MAPT n-13  Symptomatic MAPT n=20  Pre-symptomatic GRN n=125  Mild symptomatic GRN n=30  Symptomatic GRN n=43  Pre-symptomatic C9orf72 n=107  Mild symptomatic C9orf72 n=32  Symptomatic C9orf72 n=63  Controls n=298 | Cross-sectional  Case control | N/A | MRI – Cortical and subcortical volumes | Pre-symptomatic MAPT: no difference in cortical volumes; reduced subcortical volume of amygdala and hippocampus. |
| Cash et al, 2018(3) | Pre-symptomatic GRN n=65  Symptomatic GRN n=12  Pre-symptomatic C9orf72 n=40  Symptomatic C9orf72 n=25  Pre-symptomatic MAPT n=23  Symptomatic MAPT n=10  Controls n=144 | Cross-sectional  Case control | N/A | MRI - VBM | Pre-symptomatic mutation carriers: significant decrease in GM volume in the insula.  Pre-symptomatic MAPT: loss of GM volume in the orbitofrontal and medial temporal lobes. |
| Chen et al, 2019(60) | Pre-symptomatic MAPT n=12  Symptomatic MAPT n=10  Controls n=20 | Longitudinal  Case-control | Median 4 (1-5) years | MRI DTI | Pre-symptomatic MAPT: increased MD and reduced FA in the entorhinal WM associated with proximity to age of symptom onset. |
| Convery et al, 2020(5) | Pre-symptomatic C9orf72 n=73  Symptomatic C9orf72 n=31  Pre-symptomatic GRN n=104  Symptomatic GRN n=24  Pre-symptomatic MAPT n=39  Symptomatic MAPT n=10  Controls n=181 | Cross-sectional  Case control | N/A | MRI - VBM | Pre-symptomatic or symptomatic MAPT: no difference in pain perception. |
| Cury et al, 2019(6) | Pre-symptomatic GRN n=53  Pre-symptomatic C9orf72 n=34  Pre-symptomatic MAPT n=26  Controls n= 98 | Cross-sectional  Case control | N/A | MRI – large diffeomorphic deformation metric mapping | Pre-symptomatic mutation carriers: altered shape of anterior thalamus at least 5 years before expected symptom onset. |
| [Domínguez-Vivero](https://pubmed.ncbi.nlm.nih.gov/?sort=jour&term=Dom%C3%ADnguez-Vivero+C&cauthor_id=32310161) et al, 2020(61) | Pre-clinical MAPT n=12  (Pre-symptomatic n=6/12)  Controls n=44 | Cross-sectional  Case-Control | N/A | MRI – VBM, ROI analysis, WMH volume, DTI | Pre-clinical MAPT mutation carriers: reduced temporal lobe volume (left amygdala, left temporal pole), cingulate cortex (left rostral anterior cingulate gyrus, right posterior cingulate) and lingual gyrus in the occipital lobe; No DTI differences. |
| Feis et al, 2018(7) | Pre-symptomatic GRN n=35;  Pre-symptomatic C9orf72 n=72  Pre-symptomatic MAPT n=8;  Controls n = 48 | Cross-sectional  Case control | N/A | MRI – GM and WM density,DTI  rs-fMRI | Pre-symptomatic mutation carriers: best performing multimodal classification model differentiated from controls using only WM features (radial diffusivity and WM density) suggesting that earliest FTD-related changes occur in WM. |
| Fumagalli et al, 2018(9) | Pre-symptomatic GRN n= 66  Symptomatic GRN n=17  Pre-symptomatic C9orf72 n=42  Symptomatic C9orf72 n=31  Pre-symptomatic MAPT n=24  Symptomatic MAPT n=15  Controls n=148 | Cross-sectional  Case control | N/A | MRI- VBM | Pre-symptomatic MAPT: visual rating scales identified greater GM atrophy in medial temporal regions |
| Gazzina et al, 2019(10) | Pre-symptomatic GRN n=65  Pre-symptomatic C9orf72 n=31  Pre-symptomatic MAPT n=20  Controls n=113 | Longitudinal  Case-control | 4 years | MRI – GM volume | Highly educated pre-symptomatic mutation carriers: greater brain maintenance with slower GM volume loss. |
| Jiskoot et al, 2019(42) | Pre-symptomatic GRN n=30  Pre-symptomatic MAPT n=13  Controls n=30 | Longitudinal  Case-control | 4 years | MRI – VBM, TSS, DTI, ROI analysis | Pre-symptomatic MAPT: greater loss of WM integrity involving the uncinate fasciculus in the 2-years approaching phenoconversion compared to GRN carriers |
| Malpetti et al, 2021 (13) | Pre-symptomatic GRN n=142  Pre-symptomatic C9orf72 n=108  Pre-symptomatic MAPT n=54  Controls n=296 | Longitudinal  Case-control | 2-years | MRI – GM volume | Pre-symptomatic carriers: apathy progression associated with baseline low GM volume in frontal and cingulate regions. |
| Olney et al, 2020(14) | Pre-symptomatic mutation carriers n=103  Mild symptomatic carriers n=43  Dementia n=72  Controls n=102 | Cross-sectional  Case control | N/A | MRI – cortical volumes | Pre-symptomatic mutation carriers: reduced frontal and temporal volumes compared to controls.  Pre- and mild symptomatic MAPT: early insula and medial temporal involvement |
| Panman et al, 2019(15) | Pre-symptomatic GRN n=33  Pre-symptomatic MAPT n=15  Pre-symptomatic C9orf72 n=12  Controls n=53 | Longitudinal  Case-control | 2-years | MRI – VBM, cortical thickness. TBSS | Pre-symptomatic MAPT: longitudinal loss of temporal lobe GM volume and WM integrity of uncinate fasciculus. |
| Premi et al, 2017 (18) | Pre-symptomatic GRN n=61  Pre-symptomatic MAPT n=14  Pre-symptomatic C9orf72 n=33  Controls n=123 | Cross-sectional  Case control | N/A | MRI – cortical, subcortical and cerebellar volumes | Pre-symptomatic mutation carriers: education level directly affects GM volume;  TMEM106B genotype enhances the benefit of cognitive reserve on brain structure. |
| Rohrer et al, 2015(20) | Pre-symptomatic MAPT n=15  Symptomatic MAPT n=11  Pre-symptomatic GRN n=45  Symptomatic GRN n=13  Pre-symptomatic C9orf72 n=18  Symptomatic C9orf72 n=16  Controls n=102 | Cross-sectional  Case control | N/A | MRI – cortical and subcortical volumes | Pre-symptomatic MAPT: hippocampus and amygdala involvement 15-years before expected symptom onset; followed by temporal lobe 10-years before expected symptom onset; and insula 5-years before expected onset. |
| Russell et al, 2020(21) | Pre-symptomatic GRN n= 123  Symptomatic GRN n= 32  Pre-symptomatic C9orf72 n= 106  Symptomatic C9orf72 n= 53  Pre-symptomatic MAPT n= 49  Symptomatic MAPT n= 18 | Cross-sectional | N/A | MRI - VBM | Pre-symptomatic and symptomatic MAPT: social cognition correlated with left basal ganglia and left orbitofrontal cortex involvement |
| Staffaroni et al 2021(22) | Pre-symptomatic mutation carriers n=46  Symptomatic mutation carriers n=81  Controls n=101  Reference n=383 | Longitudinal | 2-years | MRI – GM volume | Pre-symptomatic mutation carriers: individualized atrophy scores predict phenoconversion, achieving 90% accuracy |
| Sudre et al, 2017(23) | Pre-symptomatic MAPT n= 8  Symptomatic MAPT n= 13  Pre-symptomatic GRN n= 25  Symptomatic GRN n= 7  Pre-symptomatic C9orf72 n= 28  Symptomatic C9orf72 n= 23  Controls n= 76 | Cross-sectional  Case control | N/A | MRI – automatic segmentation of WMH using T1 and T2 weighted images | Pre-symptomatic and symptomatic MAPT: no significant differences in WMH |
| Tavares et al, 2019(24) | Pre-symptomatic GRN n= 29  Pre-symptomatic C9orf72 n= 13  Pre-symptomatic MAPT n= 4  Controls n= 56 | Longitudinal  Case-control | 1-year | MRI – ventricular volumes | Pre-symptomatic FTD: ventricular volume difference begins on average 4 years before expected symptom onset. |
| **Functional MRI** | | | | | |
| Dopper et al, 2014(49) | Pre-symptomatic GRN n=28  Pre-symptomatic MAPT n=9  Controls n=38 | Cross-sectional  Case control | N/A | MRI – VBM, TBSS  rs-fMRI | Pre-symptomatic mutation carriers: no significant difference in GM volume  Pre-symptomatic MAPT: frontotemporal WM tracts involvement; no difference in functional connectivity. |
| Dopper et al, 2016 (50) | Pre-symptomatic MAPT n = 11  Pre-symptomatic GRN n = 23  Controls n=31 | Longitudinal  Case-control | Mean 2.2 (2.1-2.3) years | MRI – Arterial spin labelling | Pre-symptomatic MAPT: no cross-sectional differences  Pre-symptomatic mutation carriers: progressive reduced blood flow in frontal, temporal, parietal and subcortical areas |
| Feis et al, 2019(51) | Pre-symptomatic GRN n=28  Pre-symptomatic MAPT n=11  Controls = 36 | Cross-sectional  Case control | N/A | MRI – VBM, DTI, TBSS  rs-fMRI | Pre-symptomatic mutation carriers: no difference in GM volume, DTI metrics or functional connectivity. |
| Feis et al, 2019(27) | Pre-symptomatic GRN n=35  Pre-symptomatic MAPT n=8  Pre-symptomatic C9orf72 n=12  Controls = 48 | Longitudinal  Case-control | 6-years | MRI-GMD, DTI  rs-fMRI | Pre-symptomatic mutation carriers: MRI classification scores are similar to controls until close to symptom onset. |
| Mutsaerts et al, 2019(29) | Pre-symptomatic MAPT n= 18  Pre-symptomatic C9orf72 n= 34  Pre-symptomatic GRN n= 55  Controls n= 113 | Cross-sectional  Case control | N/A | MRI – Arterial spin labelling | Pre-symptomatic mutation carriers: lower blood flow in FTD regions up to 12.5 years before expected symptom onset |
| Premi et al, 2019 (30) | Pre-symptomatic C9orf72 n= 82  Pre-symptomatic GRN n= 122  Pre-symptomatic MAPT n= 45  Controls n= 223 | Cross-sectional  Case control | N/A | rs-fMRI | Pre-symptomatic MAPT: reduced meta-states numbers and overall meta-state total distance.  Pre-symptomatic mutation carriers: altered connectivity with diminished dynamic fluidity and restricted dynamic range. |
| Rittman et al, 2019(31) | Pre-symptomatic C9orf72 n= 17  Symptomatic C9orf72 n= 12  Pre-symptomatic GRN n= 40  Symptomatic GRN n= 6  Pre-symptomatic MAPT n= 13  Symptomatic MAPT n= 11  Controls n= 86 | Cross-sectional  Case control | N/A | Task-free fMRI | Pre-symptomatic mutation carriers: preserved efficient topological organisation of the brains functional network in the years leading up to expected symptom onset, despite pre-symptomatic loss of brain volume and functional connections. |
| Tsvetanov et al, 2021(33) | Pre-symptomatic C9orf72 n= 39  Pre-symptomatic GRN n= 63  Pre-symptomatic MAPT n=19  Controls n=134 | Cross-sectional  Case control | N/A | MRI – GM volume  Rs-fMRI | Pre-symptomatic mutation carriers: maintenance of functional network connectivity may enable maintenance of cognitive performance in the presence of progressive brain atrophy for years before the onset of symptoms. |
| Whitwell et al, 2011(62) | Pre-symptomatic MAPT n=8  Controls n=8  bvFTD n=21  Controls n=21 | Cross-sectional  Case-Control | N/A | fMRI | Pre-symptomatic MAPT: altered functional connectivity in the default mode network that precedes atrophy |
| **Magnetic Resonance Spectroscopy** | | | | | |
| Chen et al, 2019(63) | Pre-symptomatic MAPT n=8 | Longitudinal | Average 7.75 (4-11) years  Median 8.2 (3.8-10.7) years | Single voxel ^1^H MRS from the posterior cingulate | Pre-symptomatic MAPT: 2.09 years before symptom onset accelerated decrease in NAA/mI ratio;  1.86 years before symptom onset accelerated increase in mI/Cr ratio |
| Chen et al, 2019(64) | Pre-symptomatic MAPT n=9  Symptomatic MAPT n=10  Controls n=25 | Cross-sectional  Case-Control | N/A | Single voxel ^1^H MRS from the medial frontal lobe | Pre-symptomatic MAPT: decreased NAA/Cr and NAA/mI ratios; increased mI/Cr ratio approaching expected onset. |
| Kantarci et al, 2010(65) | Pre-symptomatic MAPT n=14  Symptomatic MAPT n=10  Controls n=24 | Cross-sectional  Case-Control | N/A | Single voxel ^1^H MRS from the posterior cingulate gyrus inferior precuneus | Pre-symptomatic MAPT: decreased NAA/mI and increased mI/Cr ratio several years before symptom onset.  NAA/Cr ratio was no different from controls. |
| **Positron Emission Tomography** | | | | | |
| Clarke et al, 2021(66) | Pre-symptomatic MAPT n=6  Controls n=12 | Cross-sectional  Case-Control | N/A | [^18^F] FDG-PET: regional standard uptake value ratios  3D T1-weighted MRI | Pre-symptomatic MAPT: reduced GM volume and [^18^F] FDG uptake in the anterior cingulate |
| Miyoshi et al, 2010 (67) | Pre-symptomatic MAPT n=3  Controls n=9 | Cross-sectional  Case-Control | N/A | [(11)C]DAA1106 PET  l-[β-^11^C]dopa PET  [(11)C]N-methylpiperidin-4-yl acetate PET  MRI | Pre-symptomatic MAPT (in some cases but not universally):  putaminal dopaminergic dysfunction, hippocampal atrophy and increased glial activation |
| Wolters et al, 2021(68) | Pre-symptomatic MAPT n= 6  Symptomatic MAPT n=3  Alzheimer’s disease n=52  Controls n=30 | Cross-sectional  Case-Control | N/A | [^18^F] flortaucipir PET scan | Pre-symptomatic MAPT: subtle elevated tau binding in the insula, frontal, parietal and medial temporal lobe.  The findings were most marked in a mutation that is associated with 3R and 4R tauopathies. |

**References**

1. Bertrand A, Wen J, Rinaldi D, Houot M, Sayah S, Camuzat A, et al. Early Cognitive, Structural, and Microstructural Changes in Presymptomatic C9orf72 Carriers Younger Than 40 Years. JAMA Neurology. 2018;75(2):236-45.

2. Bocchetta M, Todd EG, Peakman G, Cash DM, Convery RS, Russell LL, et al. Differential early subcortical involvement in genetic FTD within the GENFI cohort. NeuroImage: Clinical. 2021;30:102646.

3. Cash DM, Bocchetta M, Thomas DL, Dick KM, van Swieten JC, Borroni B, et al. Patterns of gray matter atrophy in genetic frontotemporal dementia: results from the GENFI study. Neurobiology of aging. 2018;62:191-6.

4. Caverzasi E, Battistella G, Chu SA, Rosen H, Zanto TP, Karydas A, et al. Gyrification abnormalities in presymptomatic &lt;em&gt;c9orf72&lt;/em&gt; expansion carriers. Journal of Neurology, Neurosurgery &amp;amp; Psychiatry. 2019;90(9):1005.

5. Convery RS, Bocchetta M, Greaves CV, Moore KM, Cash DM, Van Swieten J, et al. Abnormal pain perception is associated with thalamo-cortico-striatal atrophy in &lt;em&gt;C9orf72&lt;/em&gt; expansion carriers in the GENFI cohort. Journal of Neurology, Neurosurgery &amp;amp; Psychiatry. 2020;91(12):1325.

6. Cury C, Durrleman S, Cash DM, Lorenzi M, Nicholas JM, Bocchetta M, et al. Spatiotemporal analysis for detection of pre-symptomatic shape changes in neurodegenerative diseases: Initial application to the GENFI cohort. NeuroImage. 2019;188:282-90.

7. Feis RA, Bouts MJRJ, Panman JL, Jiskoot LC, Dopper EGP, Schouten TM, et al. Single-subject classification of presymptomatic frontotemporal dementia mutation carriers using multimodal MRI. NeuroImage: Clinical. 2019;22:101718.

8. Floeter MK, Bageac D, Danielian LE, Braun LE, Traynor BJ, Kwan JY. Longitudinal imaging in C9orf72 mutation carriers: Relationship to phenotype. NeuroImage Clinical. 2016;12:1035-43.

9. Fumagalli GG, Basilico P, Arighi A, Bocchetta M, Dick KM, Cash DM, et al. Distinct patterns of brain atrophy in Genetic Frontotemporal Dementia Initiative (GENFI) cohort revealed by visual rating scales. Alzheimer's research & therapy. 2018;10(1):46-.

10. Gazzina S, Grassi M, Premi E, Cosseddu M, Alberici A, Archetti S, et al. Education modulates brain maintenance in presymptomatic frontotemporal dementia. J Neurol Neurosurg Psychiatry. 2019;90(10):1124-30.

11. Le Blanc G, Jetté Pomerleau V, McCarthy J, Borroni B, van Swieten J, Galimberti D, et al. Faster Cortical Thinning and Surface Area Loss in Presymptomatic and Symptomatic C9orf72 Repeat Expansion Adult Carriers. Ann Neurol. 2020;88(1):113-22.

12. Lulé DE, Müller H-P, Finsel J, Weydt P, Knehr A, Winroth I, et al. Deficits in verbal fluency in presymptomatic <em>C9orf72</em> mutation gene carriers—a developmental disorder. Journal of Neurology, Neurosurgery &amp; Psychiatry. 2020;91(11):1195-200.

13. Malpetti M, Jones PS, Tsvetanov KA, Rittman T, van Swieten JC, Borroni B, et al. Apathy in presymptomatic genetic frontotemporal dementia predicts cognitive decline and is driven by structural brain changes. Alzheimer's & Dementia. 2021;17(6):969-83.

14. Olney NT, Ong E, Goh SM, Bajorek L, Dever R, Staffaroni AM, et al. Clinical and volumetric changes with increasing functional impairment in familial frontotemporal lobar degeneration. Alzheimers Dement. 2020;16(1):49-59.

15. Panman JL, Jiskoot LC, Bouts MJRJ, Meeter LHH, van der Ende EL, Poos JM, et al. Gray and white matter changes in presymptomatic genetic frontotemporal dementia: a longitudinal MRI study. Neurobiology of Aging. 2019;76:115-24.

16. Papma JM, Jiskoot LC, Panman JL, Dopper EG, den Heijer T, Donker Kaat L, et al. Cognition and gray and white matter characteristics of presymptomatic &lt;em&gt;C9orf72&lt;/em&gt; repeat expansion. Neurology. 2017;89(12):1256.

17. Popuri K, Dowds E, Beg MF, Balachandar R, Bhalla M, Jacova C, et al. Gray matter changes in asymptomatic C9orf72 and GRN mutation carriers. NeuroImage: Clinical. 2018;18:591-8.

18. Premi E, Grassi M, van Swieten J, Galimberti D, Graff C, Masellis M, et al. Cognitive reserve and TMEM106B genotype modulate brain damage in presymptomatic frontotemporal dementia: a GENFI study. Brain. 2017;140(6):1784-91.

19. Querin G, Bede P, El Mendili MM, Li M, Pélégrini-Issac M, Rinaldi D, et al. Presymptomatic spinal cord pathology in c9orf72 mutation carriers: A longitudinal neuroimaging study. Annals of Neurology. 2019;86(2):158-67.

20. Rohrer JD, Nicholas JM, Cash DM, van Swieten J, Dopper E, Jiskoot L, et al. Presymptomatic cognitive and neuroanatomical changes in genetic frontotemporal dementia in the Genetic Frontotemporal dementia Initiative (GENFI) study: a cross-sectional analysis. The Lancet Neurology. 2015;14(3):253-62.

21. Russell LL, Greaves CV, Bocchetta M, Nicholas J, Convery RS, Moore K, et al. Social cognition impairment in genetic frontotemporal dementia within the GENFI cohort. Cortex. 2020;133:384-98.

22. Staffaroni AM, Cobigo Y, Goh S-YM, Kornak J, Bajorek L, Chiang K, et al. Individualized atrophy scores predict dementia onset in familial frontotemporal lobar degeneration. Alzheimer's & dementia : the journal of the Alzheimer's Association. 2020;16(1):37-48.

23. Sudre CH, Bocchetta M, Cash D, Thomas DL, Woollacott I, Dick KM, et al. White matter hyperintensities are seen only in GRN mutation carriers in the GENFI cohort. Neuroimage Clin. 2017;15:171-80.

24. Tavares TP, Mitchell DGV, Coleman K, Shoesmith C, Bartha R, Cash DM, et al. Ventricular volume expansion in presymptomatic genetic frontotemporal dementia. Neurology. 2019;93(18):e1699-e706.

25. Walhout R, Schmidt R, Westeneng H-J, Verstraete E, Seelen M, van Rheenen W, et al. Brain morphologic changes in asymptomatic &lt;em&gt;C9orf72&lt;/em&gt; repeat expansion carriers. Neurology. 2015;85(20):1780.

26. Wen J, Zhang H, Alexander DC, Durrleman S, Routier A, Rinaldi D, et al. Neurite density is reduced in the presymptomatic phase of C9orf72 disease. Journal of neurology, neurosurgery, and psychiatry. 2019;90(4):387-94.

27. Feis RA, Bouts MJRJ, de Vos F, Schouten TM, Panman JL, Jiskoot LC, et al. A multimodal MRI-based classification signature emerges just prior to symptom onset in frontotemporal dementia mutation carriers. Journal of Neurology, Neurosurgery &amp;amp; Psychiatry. 2019;90(11):1207.

28. Lee SE, Sias AC, Mandelli ML, Brown JA, Brown AB, Khazenzon AM, et al. Network degeneration and dysfunction in presymptomatic C9ORF72 expansion carriers. Neuroimage Clin. 2016;14:286-97.

29. Mutsaerts HJMM, Mirza SS, Petr J, Thomas DL, Cash DM, Bocchetta M, et al. Cerebral perfusion changes in presymptomatic genetic frontotemporal dementia: a GENFI study. Brain. 2019;142(4):1108-20.

30. Premi E, Calhoun VD, Diano M, Gazzina S, Cosseddu M, Alberici A, et al. The inner fluctuations of the brain in presymptomatic Frontotemporal Dementia: The chronnectome fingerprint. NeuroImage. 2019;189:645-54.

31. Rittman T, Borchert R, Jones S, van Swieten J, Borroni B, Galimberti D, et al. Functional network resilience to pathology in presymptomatic genetic frontotemporal dementia. Neurobiol Aging. 2019;77:169-77.

32. Shoukry RS, Waugh R, Bartlett D, Raitcheva D, Floeter MK. Longitudinal changes in resting state networks in early presymptomatic carriers of C9orf72 expansions. NeuroImage: Clinical. 2020;28:102354.

33. Tsvetanov KA, Gazzina S, Jones PS, van Swieten J, Borroni B, Sanchez-Valle R, et al. Brain functional network integrity sustains cognitive function despite atrophy in presymptomatic genetic frontotemporal dementia. Alzheimer's & dementia : the journal of the Alzheimer's Association. 2021;17(3):500-14.

34. Waugh RE, Danielian LE, Shoukry RFS, Floeter MK. Longitudinal changes in network homogeneity in presymptomatic C9orf72 mutation carriers. Neurobiol Aging. 2021;99:1-10.

35. De Vocht J, Blommaert J, Devrome M, Radwan A, Van Weehaeghe D, De Schaepdryver M, et al. Use of Multimodal Imaging and Clinical Biomarkers in Presymptomatic Carriers of C9orf72 Repeat Expansion. JAMA Neurology. 2020;77(8):1008-17.

36. Malpetti M, Holland N, Jones PS, Ye R, Cope TE, Fryer TD, et al. Synaptic density in carriers of C9orf72 mutations: a [(11) C]UCB-J PET study. Ann Clin Transl Neurol. 2021;8(7):1515-23.

37. Popuri K, Beg MF, Lee H, Balachandar R, Wang L, Sossi V, et al. FDG-PET in presymptomatic C9orf72 mutation carriers. NeuroImage: Clinical. 2021;31:102687.

38. Borrego-Écija S, Sala-Llonch R, van Swieten J, Borroni B, Moreno F, Masellis M, et al. Disease-related cortical thinning in presymptomatic granulin mutation carriers. NeuroImage: Clinical. 2021;29:102540.

39. Borroni B, Alberici A, Premi E, Archetti S, Garibotto V, Agosti C, et al. Brain magnetic resonance imaging structural changes in a pedigree of asymptomatic progranulin mutation carriers. Rejuvenation Res. 2008;11(3):585-95.

40. Chen Q, Boeve BF, Senjem M, Tosakulwong N, Lesnick T, Brushaber D, et al. Trajectory of lobar atrophy in asymptomatic and symptomatic GRN mutation carriers: a longitudinal MRI study. Neurobiol Aging. 2020;88:42-50.

41. Gazzina S, Benussi A, Premi E, Paternicò D, Cristillo V, Dell'Era V, et al. Neuroanatomical Correlates of Transcranial Magnetic Stimulation in Presymptomatic Granulin Mutation Carriers. Brain Topogr. 2018;31(3):488-97.

42. Jiskoot LC, Panman JL, Meeter LH, Dopper EGP, Donker Kaat L, Franzen S, et al. Longitudinal multimodal MRI as prognostic and diagnostic biomarker in presymptomatic familial frontotemporal dementia. Brain. 2019;142(1):193-208.

43. Milanesi E, Bonvicini C, Alberici A, Pilotto A, Cattane N, Premi E, et al. Molecular signature of disease onset in granulin mutation carriers: a gene expression analysis study. Neurobiol Aging. 2013;34(7):1837-45.

44. Olm CA, McMillan CT, Irwin DJ, Van Deerlin VM, Cook PA, Gee JC, et al. Longitudinal structural gray matter and white matter MRI changes in presymptomatic progranulin mutation carriers. Neuroimage Clin. 2018;19:497-506.

45. Panman JL, Venkatraghavan V, van der Ende EL, Steketee RME, Jiskoot LC, Poos JM, et al. Modelling the cascade of biomarker changes in GRN-related frontotemporal dementia. J Neurol Neurosurg Psychiatry. 2021;92(5):494-501.

46. Paternicò D, Premi E, Gazzina S, Cosseddu M, Alberici A, Archetti S, et al. White matter hyperintensities characterize monogenic frontotemporal dementia with granulin mutations. Neurobiol Aging. 2016;38:176-80.

47. Sudre CH, Bocchetta M, Heller C, Convery R, Neason M, Moore KM, et al. White matter hyperintensities in progranulin-associated frontotemporal dementia: A longitudinal GENFI study. Neuroimage Clin. 2019;24:102077-.

48. Borroni B, Alberici A, Cercignani M, Premi E, Serra L, Cerini C, et al. Granulin mutation drives brain damage and reorganization from preclinical to symptomatic FTLD. Neurobiol Aging. 2012;33(10):2506-20.

49. Dopper EGP, Rombouts SARB, Jiskoot LC, den Heijer T, de Graaf JRA, de Koning I, et al. Structural and functional brain connectivity in presymptomatic familial frontotemporal dementia. Neurology. 2014;83(2):e19.

50. Dopper EGP, Chalos V, Ghariq E, den Heijer T, Hafkemeijer A, Jiskoot LC, et al. Cerebral blood flow in presymptomatic MAPT and GRN mutation carriers: A longitudinal arterial spin labeling study. Neuroimage Clin. 2016;12:460-5.

51. Feis RA, Bouts MJRJ, Dopper EGP, Filippini N, Heise V, Trachtenberg AJ, et al. Multimodal MRI of grey matter, white matter, and functional connectivity in cognitively healthy mutation carriers at risk for frontotemporal dementia and Alzheimer's disease. BMC neurology. 2019;19(1):343-.

52. Lee SE, Sias AC, Kosik EL, Flagan TM, Deng J, Chu SA, et al. Thalamo-cortical network hyperconnectivity in preclinical progranulin mutation carriers. Neuroimage Clin. 2019;22:101751.

53. Pievani M, Paternicò D, Benussi L, Binetti G, Orlandini A, Cobelli M, et al. Pattern of structural and functional brain abnormalities in asymptomatic granulin mutation carriers. Alzheimer's & Dementia. 2014;10(5S):S354-S63.e1.

54. Premi E, Gazzina S, Bozzali M, Archetti S, Alberici A, Cercignani M, et al. Cognitive reserve in granulin-related frontotemporal dementia: from preclinical to clinical stages. PloS one. 2013;8(9):e74762-e.

55. Premi E, Cauda F, Gasparotti R, Diano M, Archetti S, Padovani A, et al. Multimodal FMRI resting-state functional connectivity in granulin mutations: the case of fronto-parietal dementia. PloS one. 2014;9(9):e106500-e.

56. Premi E, Cauda F, Costa T, Diano M, Gazzina S, Gualeni V, et al. Looking for Neuroimaging Markers in Frontotemporal Lobar Degeneration Clinical Trials: A Multi-Voxel Pattern Analysis Study in Granulin Disease. J Alzheimers Dis. 2016;51(1):249-62.

57. Premi E, Giunta M, Iraji A, Rachakonda S, Calhoun VD, Gazzina S, et al. Dissemination in time and space in presymptomatic granulin mutation carriers: a GENFI spatial chronnectome study. Neurobiol Aging. 2021;108:155-67.

58. Caroppo P, Habert MO, Durrleman S, Funkiewiez A, Perlbarg V, Hahn V, et al. Lateral Temporal Lobe: An Early Imaging Marker of the Presymptomatic GRN Disease? J Alzheimers Dis. 2015;47(3):751-9.

59. Jacova C, Hsiung GY, Tawankanjanachot I, Dinelle K, McCormick S, Gonzalez M, et al. Anterior brain glucose hypometabolism predates dementia in progranulin mutation carriers. Neurology. 2013;81(15):1322-31.

60. Chen Q, Boeve BF, Schwarz CG, Reid R, Tosakulwong N, Lesnick TG, et al. Tracking white matter degeneration in asymptomatic and symptomatic MAPT mutation carriers. Neurobiol Aging. 2019;83:54-62.

61. Domínguez-Vivero C, Wu L, Lee S, Manoochehri M, Cines S, Brickman AM, et al. Structural Brain Changes in Pre-Clinical FTD MAPT Mutation Carriers. J Alzheimers Dis. 2020;75(2):595-606.

62. Whitwell JL, Josephs KA, Avula R, Tosakulwong N, Weigand SD, Senjem ML, et al. Altered functional connectivity in asymptomatic MAPT subjects: a comparison to bvFTD. Neurology. 2011;77(9):866-74.

63. Chen Q, Boeve BF, Tosakulwong N, Lesnick T, Brushaber D, Dheel C, et al. Brain MR Spectroscopy Changes Precede Frontotemporal Lobar Degeneration Phenoconversion in Mapt Mutation Carriers. J Neuroimaging. 2019;29(5):624-9.

64. Chen Q, Boeve BF, Tosakulwong N, Lesnick T, Brushaber D, Dheel C, et al. Frontal lobe (1)H MR spectroscopy in asymptomatic and symptomatic MAPT mutation carriers. Neurology. 2019;93(8):e758-e65.

65. Kantarci K, Boeve BF, Wszolek ZK, Rademakers R, Whitwell JL, Baker MC, et al. MRS in presymptomatic MAPT mutation carriers: a potential biomarker for tau-mediated pathology. Neurology. 2010;75(9):771-8.

66. Clarke MTM, St-Onge F, Beauregard JM, Bocchetta M, Todd E, Cash DM, et al. Early anterior cingulate involvement is seen in presymptomatic MAPT P301L mutation carriers. Alzheimers Res Ther. 2021;13(1):42.

67. Miyoshi M, Shinotoh H, Wszolek ZK, Strongosky AJ, Shimada H, Arakawa R, et al. In vivo detection of neuropathologic changes in presymptomatic MAPT mutation carriers: A PET and MRI study. Parkinsonism & Related Disorders. 2010;16(6):404-8.

68. Wolters EE, Papma JM, Verfaillie SCJ, Visser D, Weltings E, Groot C, et al. [(18)F]Flortaucipir PET Across Various MAPT Mutations in Presymptomatic and Symptomatic Carriers. Neurology. 2021;97(10):e1017-e30.
